# FtsZ forms biomolecular condensates in a polar-growing Alphaproteobacterium

**DOI:** 10.1128/mbio.00494-26

**Published:** 2026-04-15

**Authors:** Todd A. Cameron, Miguel Ángel Robles-Ramos, Lorenzo Suigo, Silvia Zorrilla, William Margolin

**Affiliations:** 1 University of Texas Health Science Center at Houston 12340, Houston, Texas, USA; 2 Centro de Investigaciones Biológicas Margarita Salas, Consejo Superior de Investigaciones Científicas (CSIC) 383204, Madrid, Spain; University of California, Berkeley, California, USA

**Keywords:** FtsZ, *Agrobacterium tumefaciens*, *Caulobacter crescentus*, biomolecular condensates, cell cycle, alphaproteobacteria, fluorescence microscopy

## Abstract

The conserved tubulin homolog FtsZ assembles into GTP-dependent protofilaments that organize the bacterial cell division machinery. Purified *Escherichia coli* FtsZ (FtsZ_Ec_) can form biomolecular condensates in the absence of GTP in macromolecular crowding conditions, but FtsZ condensates have not yet been confirmed *in vivo*. FtsZ from the Alphaproteobacterium *Agrobacterium tumefaciens* (FtsZ_At_) localizes to distinct polar foci during growth and contains an intrinsically disordered region much longer than FtsZ_Ec_, suggesting a greater tendency to form condensates. Here, we show that purified FtsZ_At_ readily formed dynamic condensates *in vitro* under molecular crowding conditions, and the addition of GTP induced the formation of filaments radiating from the FtsZ_At_ droplets. Notably, FtsZ_At_ formed similar foci and filaments *in vivo* that dynamically assembled and coalesced, supported by fluorescence recovery after photobleaching analysis of FtsZ_At_ and another Alphaproteobacterial FtsZ. These results suggest that FtsZ_At_ forms polar condensates in *A. tumefaciens* cells, perhaps to sequester FtsZ_At_ before cell division as an adaptation to slow generation times.

**IMPORTANCE:**

Alphaproteobacteria are highly diverse; however, most orthologs of the essential cell division protein FtsZ from this class feature an unusually long intrinsically disordered internal domain compared with other bacterial FtsZs. As proteins harboring such domains have an increased tendency to assemble into phase-separated biomolecular condensates, we investigated the condensate-forming properties of Alphaproteobacterial FtsZ proteins *in vivo* and *in vitro*. We found that *Agrobacterium tumefaciens* FtsZ has a strong tendency to form condensates *in vitro* and *in vivo* as part of its cell cycle, as does FtsZ of *Caulobacter crescentus,* a distantly related Alphaproteobacterium. This behavior contrasts with FtsZ from *Escherichia coli,* which has a much shorter internal disordered domain and forms condensates in combination with partner proteins under more stringent conditions. Our results suggest that Alphaproteobacterial FtsZs form condensates during normal growth, perhaps serving as part of a conserved mechanism of cell cycle control in this class of bacteria.

## INTRODUCTION

Biomolecular condensates form when proteins, driven by multivalent interactions, undergo liquid-liquid phase separation and form distinct droplets of concentrated biomolecules that partially exclude the aqueous solvent (1, 2). Condensate formation can be driven by electrostatic or hydrophobic interactions and is facilitated by protein domains that are intrinsically disordered regions (IDRs), bind nucleic acids, or promote oligomerization (3, 4). In bacteria, condensates can function as transient organelles that either concentrate or sequester interacting proteins and substrates, depending on the physical properties of each component. As such, condensates are increasingly recognized to serve roles in a wide variety of microbial processes, including RNA metabolism, signal transduction, DNA replication, and establishing cell polarity (5, 6).

Condensates have also been linked to the regulation of cell division in several bacteria. In most bacteria, cell division relies on the organized assembly of FtsZ, the bacterial homolog of tubulin, into GTP-dependent treadmilling polymers at midcell (7). The formation of these FtsZ polymer filaments, which serve as scaffolds for an assortment of peptidoglycan synthases and other proteins necessary to complete the complex process of cell septation, is regulated by a variety of mechanisms across bacterial species, including several known to involve condensates (8). In *Myxococcus xanthus*, PomY condensates, in conjunction with PomX and PomZ, attract FtsZ proteins to the midcell and nucleate their polymerization as a form of positive regulation (9, 10). Likewise, *in vitro* experiments with *E. coli* FtsZ (FtsZ_Ec_) have shown that under macromolecular crowding cytomimetic conditions it can form condensates on its own or in cooperation with either of two FtsZ-regulatory proteins, SlmA or MatP (11–13). In all cases, FtsZ_Ec_ condensates form in the absence of GTP, suggesting that their formation may primarily arise under starvation conditions or bacterial quiescence. Although FtsZ_Ec_ does not form visible foci or aggregates during normal growth, FtsZ_Ec_ foci often accumulate at cell poles in quiescent *E. coli* cells, suggesting that these condensates may form *in vivo* under certain conditions (14).

Two factors likely contribute to the ability of FtsZ to form condensates *in vitro*. One is the tendency of FtsZ_Ec_ to self-associate, forming polymers in the presence of GTP and oligomers in its absence (15). In addition to the strong longitudinal interactions that enable polymerization in the presence of GTP, FtsZ is also capable of weaker lateral interactions between FtsZ polymers (16), indicating that FtsZ monomers can readily develop multivalent interactions with each other. Another is the flexible linker domain, an IDR at the C-terminus of FtsZ. Although removal of the 50 amino acid linker from FtsZ_Ec_ still permits the formation of condensates *in vitro*, these condensates are smaller and assemble more slowly than with the wild-type protein, indicating that the linker is nonetheless important for modulating condensate formation and behavior (12).

**Fig 1:**
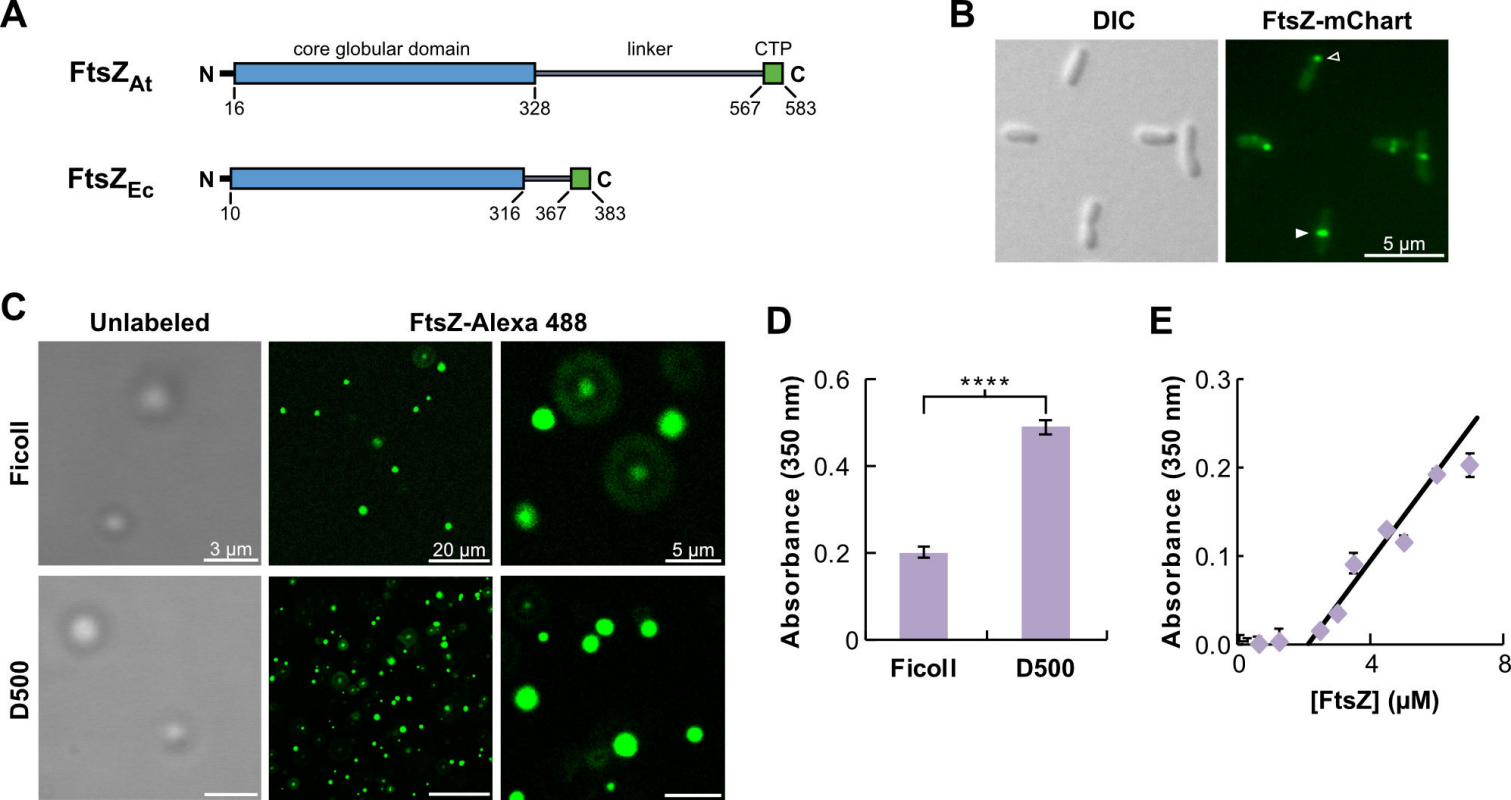
FtsZ_At_ assembles into condensates under crowding conditions. (**A**) FtsZ protein domain diagrams for *A. tumefaciens* FtsZ (FtsZ_At_) and *E. coli* FtsZ (FtsZ_Ec_). The conserved core globular domain is highlighted in blue, the linker in gray, and the C-terminal peptide (CTP) in green. (**B**) DIC and fluorescence microscopy of *A. tumefaciens* strain WM7687 expressing FtsZ-mChartreuse (FtsZ_At_-mChart). FtsZ_At_-mChart localizes to both midcell rings (solid arrowhead) and polar foci (hollow arrowhead). (**C**) Purified FtsZ_At_ (7 μM) formed putative biomolecular condensates in solutions with Ficoll 70 (Ficoll) or dextran 500 (D500) as crowder agents. FtsZ_At_ labeled with green fluorescent Alexa Fluor 488 (FtsZ-Alexa 488) added as a tracer (0.5 μM), also localized to condensates; images at two different magnifications are shown. (**D**) Turbidity of FtsZ_At_ (7 μM) condensates formed in Ficoll or D500 was assessed by absorbance at 350 nm. Asterisks indicate two-sample *t*-test significance level: ****P < 0.0001. (**E**) Variation of turbidity with FtsZ_At_ concentration in Ficoll. Saturation concentration (C_sat_), calculated from the intersection of the fitted line with the *x*-axis, was 2.12 ± 0.05 µM. *In vitro* condensate experiments were performed in 50 mM Tris-HCl, pH 7.5, 100 mM KCl, and 1 mM MgCl_2_ with the specified crowder (150 g/L) in the absence of GTP. Samples were incubated for 30 min before initial visualization of condensates.

Among the FtsZ proteins found across different bacterial lineages, those of the class Alphaproteobacteria possess especially long linker regions (17, 18). For example, FtsZ from the well-characterized *Caulobacter crescentus* has a linker of more than 170 amino acids, and FtsZ from another well-known Alphaproteobacterium, *Agrobacterium tumefaciens*, has a ~240 residue linker, almost five times the length of the FtsZ_Ec_ linker (Fig. 1A). These long FtsZ linkers are predominant throughout most of the highly diverse Alphaproteobacteria orders (Fig. S1). Given the findings with FtsZ_Ec_, we hypothesized that such extended linkers might significantly enhance FtsZ condensate formation. Intriguingly, both *A. tumefaciens* and *C. crescentus* FtsZ proteins can localize as foci at cell poles prior to the assembly of a midcell Z-ring (Fig. 1B) (19–23). Polar FtsZ foci in *A. tumefaciens* were observed by ectopic expression of FtsZ-GFP at 10% of native FtsZ levels or by immunostaining of natively expressed FtsZ (21). Similarly, polar foci of FtsZ in *C. crescentus* were observed from FtsZ expressed from the native *ftsZ* locus (19, 20). Therefore, in both species, FtsZ polar focus formation is unlikely a result of overexpression. This distinctive localization pattern at native or near-native protein levels suggests Alphaproteobacteria may form FtsZ condensates as part of their cell cycle.

In this study, we have examined in detail the ability of *A. tumefaciens* FtsZ (FtsZ_At_) to form condensates *in vitro* and *in vivo*. We found that purified FtsZ_At_ readily formed dynamic condensates under molecular crowding conditions. Addition of GTP induced the formation of filaments radiating from FtsZ_At_ condensates, and similar patterns of foci and filaments of FtsZ_At_ were observed when expressed ectopically in *E. coli* cells. FtsZ_At_ foci formed *in vivo* also exhibited dynamic coalescence in timelapse microscopy studies and demonstrated active subunit exchange when subjected to fluorescence recovery after photobleaching (FRAP) analysis. Together, these results suggest that FtsZ in *A. tumefaciens*, and likely other Alphaproteobacteria, readily phase-separates into biomolecular condensates, possibly reflecting a conserved mechanism to sequester FtsZ in between cell division events.

## RESULTS

### Purified FtsZ_At_ forms phase-separated droplets in crowded cytomimetic conditions

Although the *A. tumefaciens* strain C58 (syn. *Rhizobium radiobacter* strain C58, syn. *Agrobacterium fabrum* strain C58) genome encodes three FtsZ homologs, only one, Atu2086, is essential for viability and independently localizes to the midcell (24). As Atu2086 (FtsZ_At_) is also the only *A. tumefaciens* FtsZ paralog encoding a full-length protein containing a linker and CTP domain, we focused on this FtsZ paralog for the present study.

To investigate the potential of FtsZ_At_ to form condensates, we first examined its properties *in vitro*. Untagged, full-length FtsZ_At_ was purified and exhibited concentration-dependent GTP polymerization with an estimated critical concentration of 3 µM (Fig. S2A) and GTPase activity rates of 3–5 mol P_i_ · (mol FtsZ)^−1^ · min^−1^ (Fig. S2D). These results are consistent with the previously reported characterization of FtsZ_At_, which found GTPase activity rates of 4.7 ± 0.2 mol P_i_ · (mol FtsZ)^−1^ · min^−1^ and a critical concentration between 2 and 4 μM FtsZ_At_ (24). Addition of GTP to 7 μM FtsZ_At_ also led to the formation of long polymer filaments as detected by transmission electron microscopy (Fig. S2B) and turbidity measurements (Fig. S2C), consistent with typical *in vitro* characteristics of purified FtsZ protein from *A. tumefaciens* and *E. coli* (24–27).

However, when either 150 g/L Ficoll 70 or dextran 500 was added to simulate a crowded cellular environment, 7 μM FtsZ_At_ formed round structures—putative biomolecular condensates—that were visible by light microscopy (Fig. 1C, “Unlabeled”). These structures formed in buffer conditions similar to those used for FtsZ polymerization, 50 mM Tris-HCl, pH 7.5, with 100 mM KCl and 1 mM MgCl_2_, except for the addition of a molecular crowder and in the absence of GTP. To confirm that these structures were composed of FtsZ_At_ protein, the experiment was repeated with the addition of a small amount (0.5 μM) of FtsZ_At_ labeled with Alexa Fluor 488 as a fluorescent tracer. Confocal fluorescence microscopy revealed the formation of multiple round structures in which labeled FtsZ_At_ accumulated (Fig. 1C).

Condensate formation was also assessed by measuring the turbidity of FtsZ_At_ solutions at 350 nm. Turbidity levels of FtsZ_At_ were elevated in the presence of each crowder, further supporting the formation of higher-level structures in the presence of molecular crowders (Fig. 1D). A hallmark of biomolecular condensation is the existence of a threshold concentration (C_sat_) above which phase separation occurs. To determine the minimal concentration of FtsZ required to form condensates in the presence of 150 g/L Ficoll, turbidity was also measured for solutions containing FtsZ_At_ concentrations from 0 to 7 μM (Fig. 1E). A linear fit of the resulting data indicated a C_sat_ of 2.12 ± 0.05 µM for FtsZ_At_. Together, these experiments indicate that FtsZ_At_ forms condensates under molecular crowding conditions with protein concentrations and buffer characteristics that are physiologically relevant and consistent with the formation of FtsZ filaments upon addition of GTP.

To understand the impacts of crowding effects and ionic strength on FtsZ_At_ condensate formation, turbidity was examined under varying crowder and salt concentrations. Crowding strongly affected FtsZ_At_ condensate formation (Fig. S3A), with the turbidity of the samples nearly doubling at 200 g/L Ficoll compared to 150 g/L Ficoll. Visual examination of condensate formation by confocal microscopy correlated well with turbidity measurements, with nearly no droplets visible at 100 g/L compared to higher concentrations of crowder (Fig. S3A). In contrast, increasing the solution ionic strength from 100 mM KCl had only a minor effect, with both 300 mM and 500 mM KCl solutions reducing turbidity by only one-third (Fig. S3B). This partial effect suggests that non-ionic interactions play a substantial role in the formation of FtsZ_At_ condensates.

### Condensates formed by purified FtsZ_At_ are dynamic

**Fig 2:**
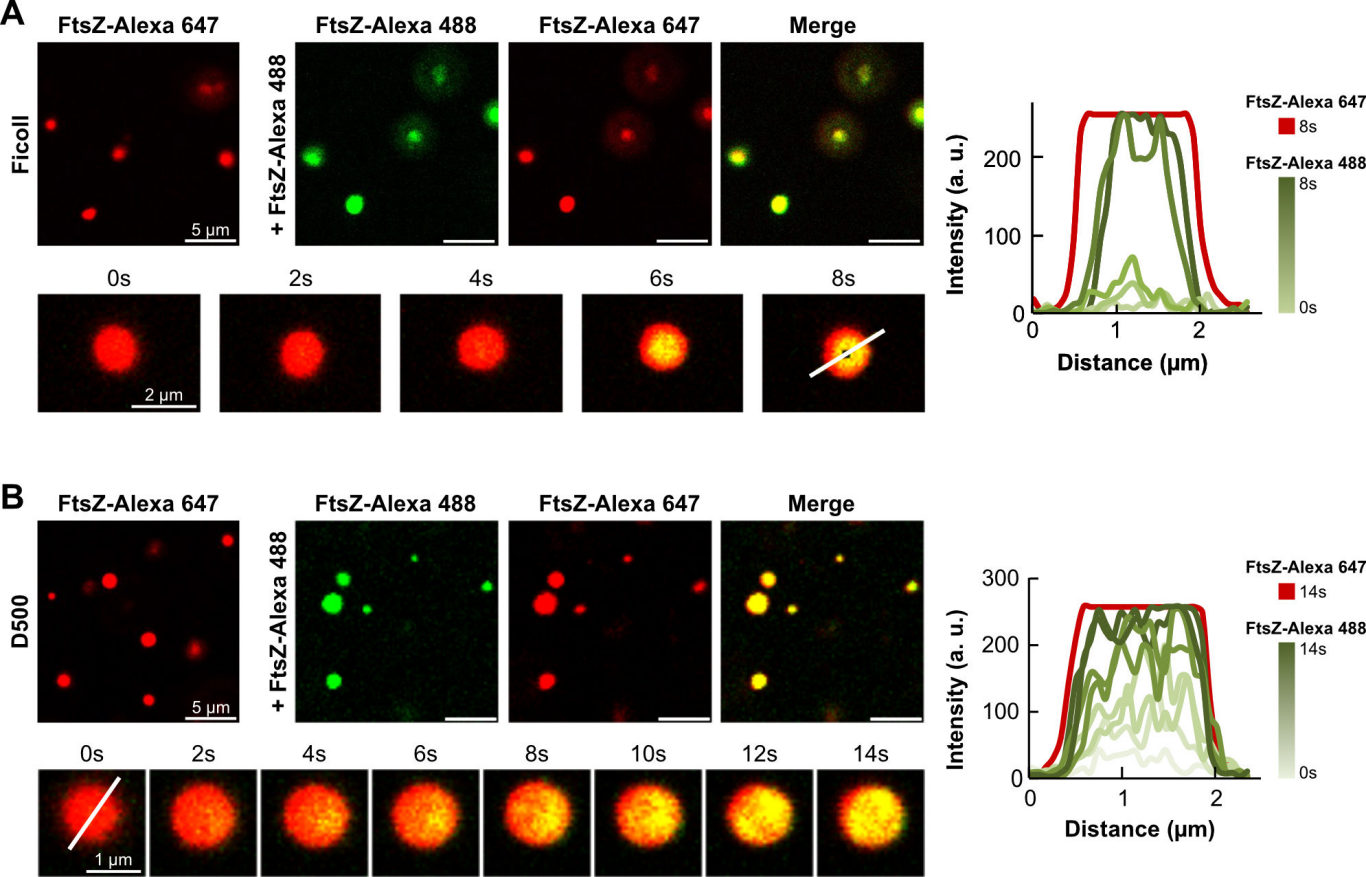
FtsZ_At_ condensates are dynamic. (**A and B**) Top: fluorescence micrographs of capture experiments consisting of the addition of fresh FtsZ_At_-Alexa 488 (0.5 μM, green) over preformed condensates containing unlabeled FtsZ_At_ (7 μM) and FtsZ_At_ labeled with Alexa Fluor 647 (FtsZ_At_-Alexa 647, 0.5 μM, red) as a tracer in solutions with (**A**) Ficoll or (**B**) D500 as crowders. Bottom: stepwise capture of added FtsZ_At_-Alexa 488 by individual condensates. Numbers above the images indicate elapsed time in seconds. Right: corresponding intensity profiles measured across the condensate (white line) at each time point. The profile in the red channel at 8 s (**A**) or 14 s (**B**) is shown as a reference. Condensate experiments were prepared as described for Fig. 1.

We next sought to characterize the extent to which these FtsZ_At_ condensates would allow dynamic exchange of protein. To test this, we utilized a two-color capture assay previously used to assess dynamic interchange of FtsZ_Ec_ and other condensates *in vitro* (11, 12, 28). First, FtsZ_At_ condensates were formed in the presence of 0.5 μM FtsZ_At_-Alexa 647 (red) and imaged by confocal microscopy (Fig. 2A and B, left). Subsequent addition of 0.5 μM FtsZ_At_-Alexa 488 (green) and observation by confocal microscopy revealed uniform incorporation of FtsZ_At_-Alexa 488 into the pre-existing condensates. Timelapse microscopy and quantification of line profiles of individual condensates further demonstrated that this incorporation occurred rapidly over the course of a few seconds in the presence of both Ficoll and D500 (Fig. 2A and B, bottom and right). These results indicate that FtsZ_At_ condensates, much like those formed by FtsZ_Ec_, are dynamic.

**Fig 3:**
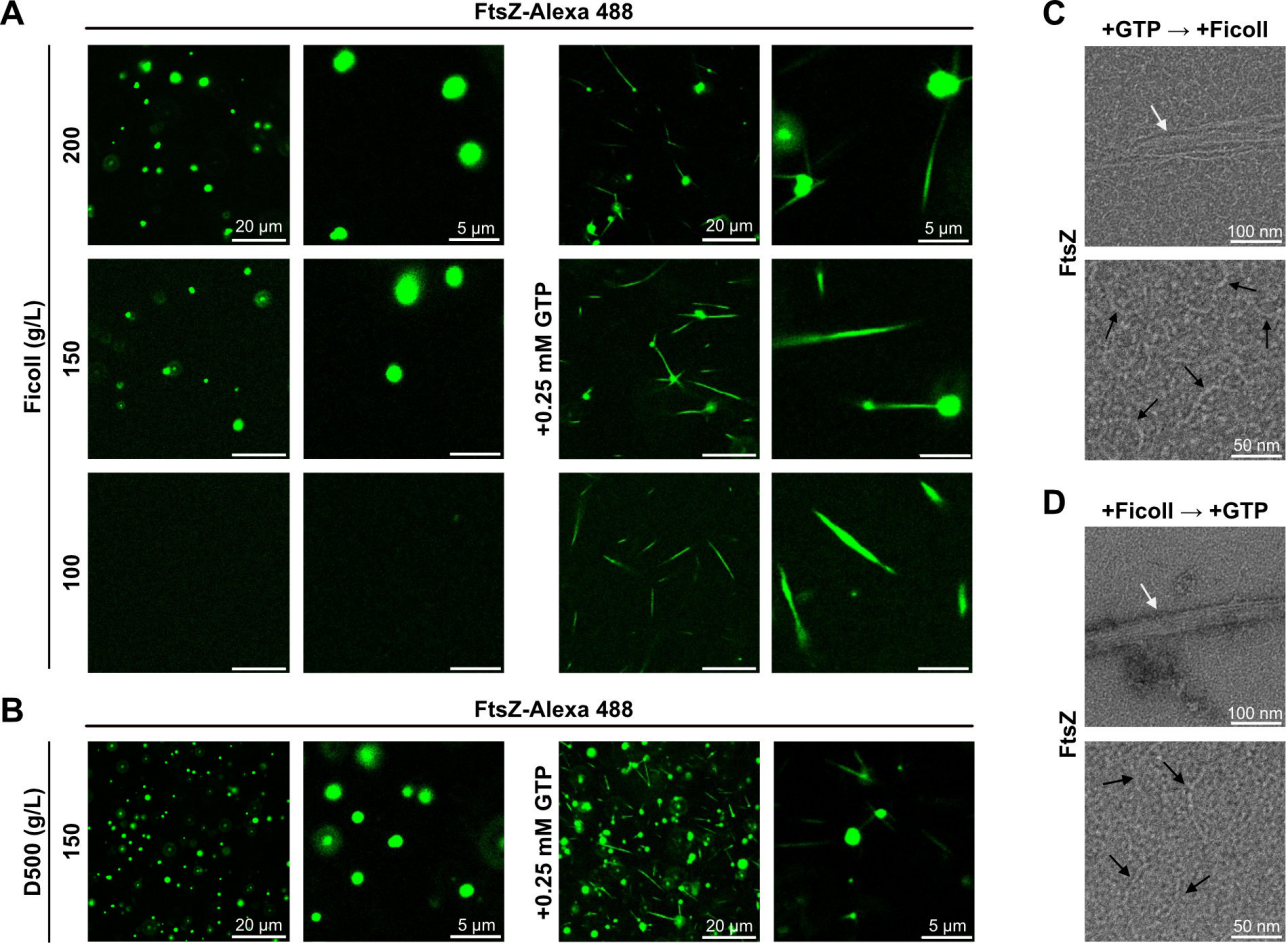
FtsZ_At_ condensate behavior in response to GTP addition. (**A**) Fluorescence micrographs of FtsZ_At_ before (left) and after (right) addition of 0.25 mM GTP with Ficoll concentrations of 100, 150, or 200 g/L demonstrate the formation of FtsZ filaments in all conditions. Images at two different magnifications are shown. (**B**) Fluorescence micrographs of FtsZ_At_ condensates formed in the presence of D500 before (left) and after (right) the addition of 0.25 mM GTP. The intensity of the leftmost image after GTP addition was enhanced to better reflect bundle formation. (**C and D**) Transmission electron microscopy images of FtsZ_At_ samples in Ficoll and 2 mM GTP. GTP was added (**C**) immediately before Ficoll addition or (**D**) 30 min after incubation with Ficoll to form condensates. In both cases, samples were incubated for 5 min before diluting 1:5 immediately prior to uranyl acetate staining. The bottom image in each panel is a magnification of the top image. The black arrows mark thin filaments, while the white arrows point to a bundle of FtsZ_At_ filaments. Condensate experiments were prepared as described for Fig. 1, with the addition of GTP as described.

Since FtsZ assembles into dynamic filaments in the presence of GTP, we next explored how FtsZ_At_ condensate formation might affect FtsZ_At_ polymerization. Samples were incubated with Ficoll at concentrations sufficient (150 or 200 g/L) or insufficient (100 g/L) to promote condensate formation (Fig. 3A, left). Upon the addition of 0.25 mM GTP, similar filaments were formed under all conditions (Fig. 3A, right); however, in the presence of condensates, these filaments sometimes radiated directly from the condensates (Fig. 3A, middle and top right). Addition of GTP to condensates formed with D500 also produced filaments radiating from condensates (Fig. 3B). Transmission electron microscopy images of FtsZ_At_ after the addition of GTP followed by Ficoll (Fig. 3C), or Ficoll followed by GTP (Fig. 3D), show polymer bundles, along with thinner polymers not resolved in fluorescence micrographs in both conditions. Overall, these data demonstrate that FtsZ_At_ protein in *in vitro* condensates is dynamic and can assemble into polymers in response to GTP.

### FtsZ_At_ forms dynamic foci *in vivo* consistent with condensates

**Fig 4:**
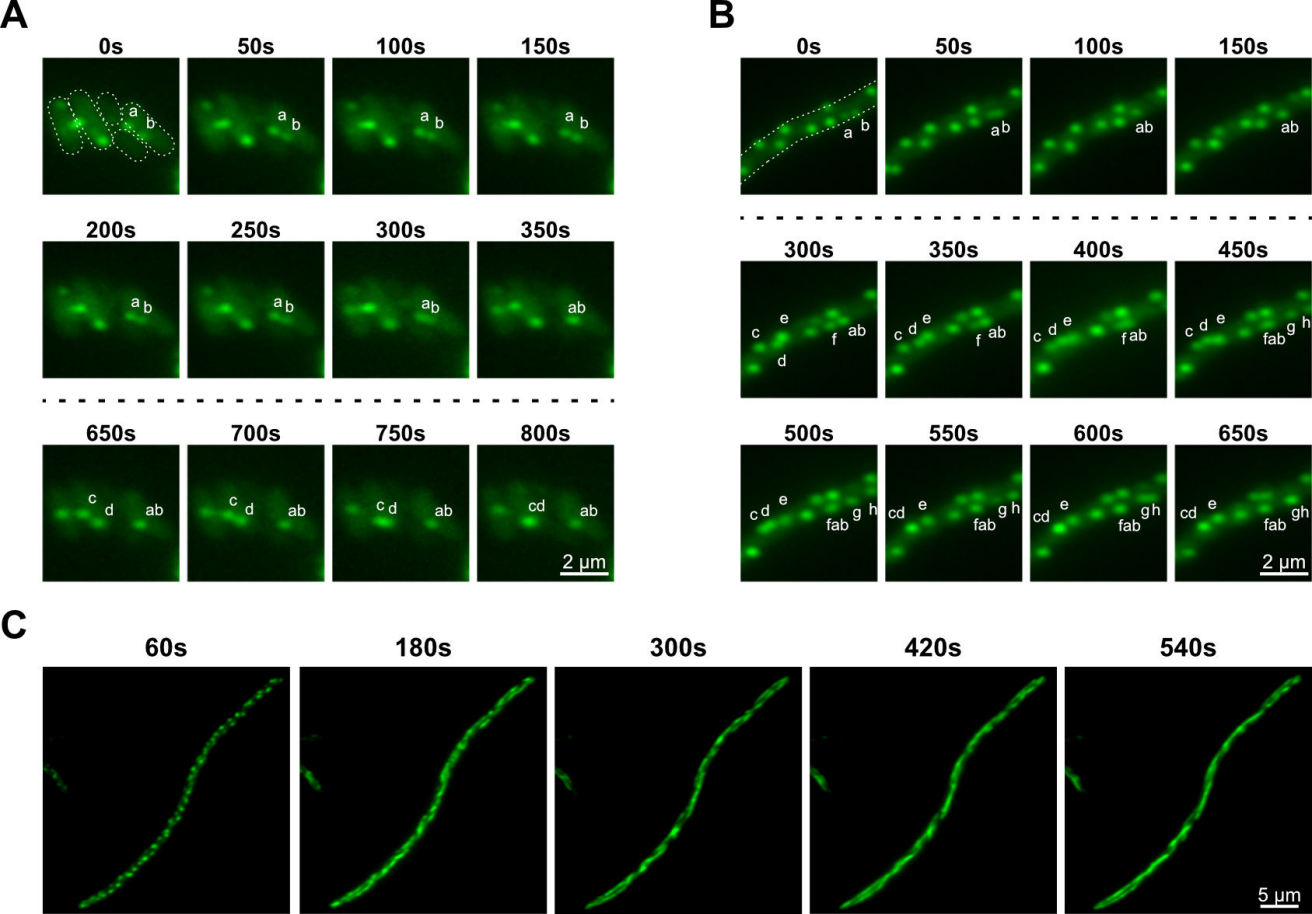
FtsZ_At_ forms dynamic foci *in vivo*. Timelapse fluorescence microscopy of *A. tumefaciens* or *E. coli* cells expressing FtsZ_At_-mChart produced foci exhibiting dynamic behaviors. (**A**) *A. tumefaciens* strain WM7687 was induced with 1 mM IPTG for 15 h, and cells were mounted on M9-glucose agarose pads. Foci a and b merged to become ab, and foci c and d merged to become cd. Dotted lines indicate cell boundaries. (**B**) Expression of FtsZ_At_-mChart from *E. coli* strain WM7689 was induced by 100 μM IPTG for 2 h; then, the cells were resuspended in water and mounted on water-agarose pads for viewing. Foci a and b merged to become ab, then later merged with focus f to become fab. Foci c and d merged to become cd. Focus g formed and merged with h to become gh. (**C**) *E. coli* cells prepared as in panel **B** but mounted on agarose pads prepared with 50% strength M9-glucose. Foci transitioned into long, dynamic filaments. Relative elapsed time is indicated above the images in panels **A** and **B**, and total elapsed time after mounting on the agarose pad is indicated for images in panel **C**.

Next, we asked whether the characteristics of FtsZ_At_ foci *in vivo* were consistent with condensates observed *in vitro*. For this, we constructed a C-terminal FtsZ_At_-mChartreuse (FtsZ_At_-mChart) fusion, using a recently developed green fluorophore with enhanced photostability (29). This fusion was expressed from an IPTG-inducible plasmid in *A. tumefaciens* strain C58, revealing patterns of polar and midcell foci (Fig. 1B) consistent with earlier studies of FtsZ_At_ (21). To observe interactions between multiple FtsZ_At_-mChart foci, cells were induced for a longer period and imaged by timelapse widefield fluorescence microscopy. Intriguingly, when cells with multiple foci were examined over the course of several minutes, FtsZ_At_ foci were often observed migrating and coalescing together into new stable foci (Fig. 4A; Movie S1). This behavior is highly reminiscent of behaviors expected of biomolecular condensates (5).

To better isolate the behaviors of FtsZ_At_-mChart from native interacting proteins, we introduced the plasmid carrying FtsZ_At_-mChart into *E. coli* strain WM1074, an MG1655 derivative. At low induction levels in *E. coli*, FtsZ_At_-mChart localized to midcell rings, likely by co-polymerizing into the native *E. coli* Z-ring (Fig. S4B), similar to what was described previously (30). However, at high expression levels, FtsZ_At_-mChart localized to distinct structures, ranging from extended lateral filaments to discrete foci, which were dependent on different media and mounting conditions (Fig. S4A). Higher expression levels of FtsZ_At_-mChart in *E. coli* also resulted in cell elongation, likely due to titration of native *E. coli* FtsZ away from division sites. Although overexpression of FtsZ_Ec_-GFP in *E. coli* also caused cell elongation, FtsZ_Ec_-GFP did not form the same patterns of foci or lateral filaments as FtsZ_At_-mChart (Fig. S4C and D).

**Fig 5:**
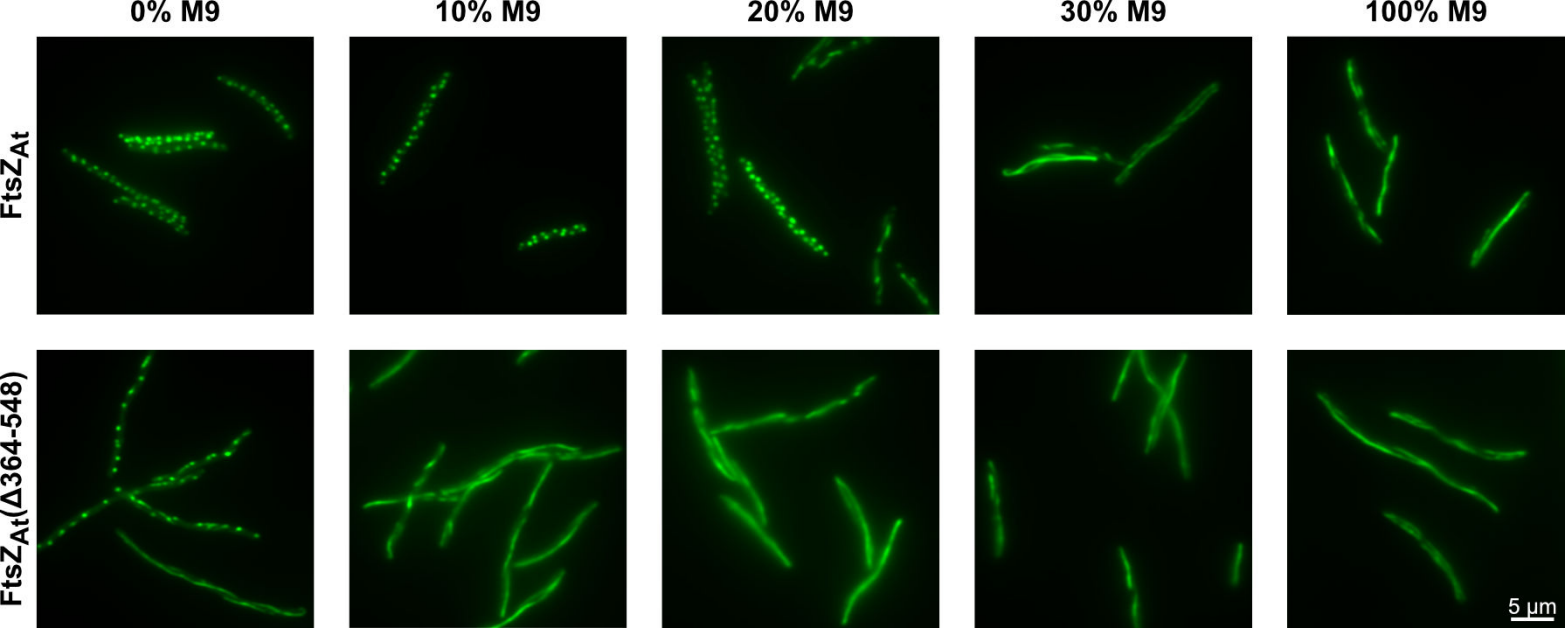
FtsZ_At_ linker truncation reduces focus formation in *E. coli*. Localization of full-length FtsZ_At_-mChart or the FtsZ_At_(Δ364-548)-mChart linker truncation expressed at high levels with 100 μM IPTG in the *E. coli* strain WM1074 background. After induction, cells were washed and mounted on agarose pads in different dilutions of M9-glucose media in water. Compared to the full-length protein, the FtsZ_At_ linker truncation significantly resisted conversion to foci at dilute concentrations of M9-glucose.

Curiously, although FtsZ_At_-mChart in cells grown in lysogeny broth (LB) media and imaged in liquid LB formed foci or mixed foci and filaments, mounting the same cells on agarose pads prepared with LB media or M9-glucose media resulted in only FtsZ_At_-mChart filaments. Similar filaments were also obtained when clarified, spent LB media were used to prepare LB-agarose pads (data not shown), suggesting that this effect was not due to short-term changes in nutrient availability. Conversely, when *E. coli* cells were suspended in plain water or mounted on agarose pads prepared with water, FtsZ_At_-mChart formed numerous foci resembling those found in *A. tumefaciens* (Fig. 4B and 5; Fig. S4A). Timelapse microscopy of cells mounted on water-agarose pads showed FtsZ_At_-mChart foci repeatedly coalescing and forming (Fig. 4B; Movie S2), replicating behaviors observed in *A. tumefaciens*.

Next, we imaged the transition from FtsZ_At_-mChart foci to filaments by placing cells suspended in water onto agarose pads prepared with M9-glucose media. However, the use of full-strength M9 resulted in complete conversion to filaments before cells could be imaged. In contrast, mounting cells on an agarose pad prepared with a 50% dilution of M9-glucose in water sufficiently slowed the process, revealing FtsZ_At_-mChart foci that gradually elongated into extended lateral filaments over the course of several minutes (Fig. 4C; Movie S3). Together, these experiments show that FtsZ_At_-mChart contained in foci remains fully capable of polymerization, consistent with our *in vitro* findings demonstrating GTP-induced FtsZ polymerization under condensate-forming conditions.

### Deletion of the IDR reduces FtsZ_At_ condensate formation *in vivo*

We next tested what role, if any, the extended FtsZ_At_ linker might play in the formation of these FtsZ_At_-mChart foci. A truncation of the FtsZ linker, FtsZ_At_(Δ364-548)-mChart, was constructed to retain the first 35 and last 15 amino acids of the original linker sequence, forming a 50 amino acid linker, the same length as the native FtsZ_Ec_ linker. Like the full-length FtsZ_At_-mChart protein, when expressed at high levels in *E. coli*, the FtsZ_At_ linker truncation also formed dynamic filaments when mounted on M9-agarose pads. However, the FtsZ_At_ linker truncation strongly resisted conversion to foci when mounted on agarose pads prepared with increasingly dilute concentrations of M9 media (Fig. 5). While the full-length protein formed foci when M9-glucose was diluted below 30%, the FtsZ_At_ linker truncation did not completely convert to foci even when mounted on water-agarose pads. This demonstrates that the extended linker region of FtsZ_At_ expands the range of conditions in which FtsZ foci form *in vivo* in comparison to the truncated linker.

### Co-localization of FtsZ_At_ with the known condensate-forming protein PopZ *in vivo*

In Alphaproteobacteria, cell polarity is established in part by the PopZ protein, which forms well-characterized, dense condensates at the cell poles of *C. crescentus* that interact with other proteins and condensates present at the cell poles (31, 32). Previous studies have indicated that PopZ_At_ localizes to the *A. tumefaciens* growth pole and further affects the localization of the Z-ring when cells transition from polar growth to cell division (23, 33, 34). Given the potential for an interaction between PopZ_At_ and FtsZ_At_, we generated a red fluorescent mLychee-PopZ_At_ fusion and co-expressed it with FtsZ_At_-mChart in *E. coli*. Although mLychee-PopZ_At_ formed discrete foci in cells, it did not recruit FtsZ_At_-mChart when the latter was expressed at low induction levels (Fig. S5A). With higher expression of FtsZ_At_-mChart, both proteins frequently appeared to nearly or completely co-localize in cells mounted on water-agarose pads (Fig. S5A). However, when cells were treated with Hoechst to stain nucleoids, it became apparent that both mLychee-PopZ_At_ and FtsZ_At_-mChart foci strongly co-localized to nucleoid-free regions of these cells (Fig. S5B). Although this suggests their apparent co-localization was likely incidental, the localization of FtsZ_At_-mChart foci to nucleoid-free regions nonetheless mimics the behavior of the PopZ condensates and supports the idea that FtsZ_At_-mChart foci also represent large macromolecular structures.

### Additional evidence for FtsZ_At_ condensates *in vivo*

The aliphatic alcohol 1,6-hexanediol is frequently used to test potential condensates *in vivo* due to its ability to disrupt weak hydrophobic interactions. However, hexanediol can also cause broad physiological changes—including perturbations of kinases, phosphatases, transcription, and membrane integrity—that complicate interpretation of its effects (35–37). Indeed, when *A. tumefaciens* cells expressing FtsZ_At_-mChart were treated with 2% hexanediol, a concentration lower than what typically disrupts condensates, midcell localization was eliminated, whereas polar localization remained unaffected (Fig. S6A). Low levels of hexanediol may selectively interfere with FtsZ_At_ ring formation, perhaps by disrupting FtsZ_At_ polymerization or the functions of FtsZ_At_ membrane tethering proteins like FtsA and FzlC (38, 39). Although the exact nature of this selective disruption of FtsZ_At_ at the midcell is unclear, this difference suggests that polar FtsZ_At_ foci utilize a different localization mode than FtsZ_At_ polymers in midcell Z-rings. When hexanediol concentrations were increased to 6% or 8%, polar localization of FtsZ_At_ grew increasingly aberrant or diffuse, and these effects were largely temporary, as normal localization could still be recovered after removal of hexanediol (Fig. S6A through C). Although these results suggest FtsZ_At_ is sensitive to hexanediol, hexanediol treatment of *in vitro* FtsZ_At_ condensates formed with Ficoll or dextran did not result in any apparent disruption or decrease in condensates (Fig. S6D and E). It is unclear if the *in vivo* environment renders FtsZ_At_ more sensitive to hexanediol or if hexanediol indirectly affects physiological parameters important for condensate formation.

**Fig 6:**
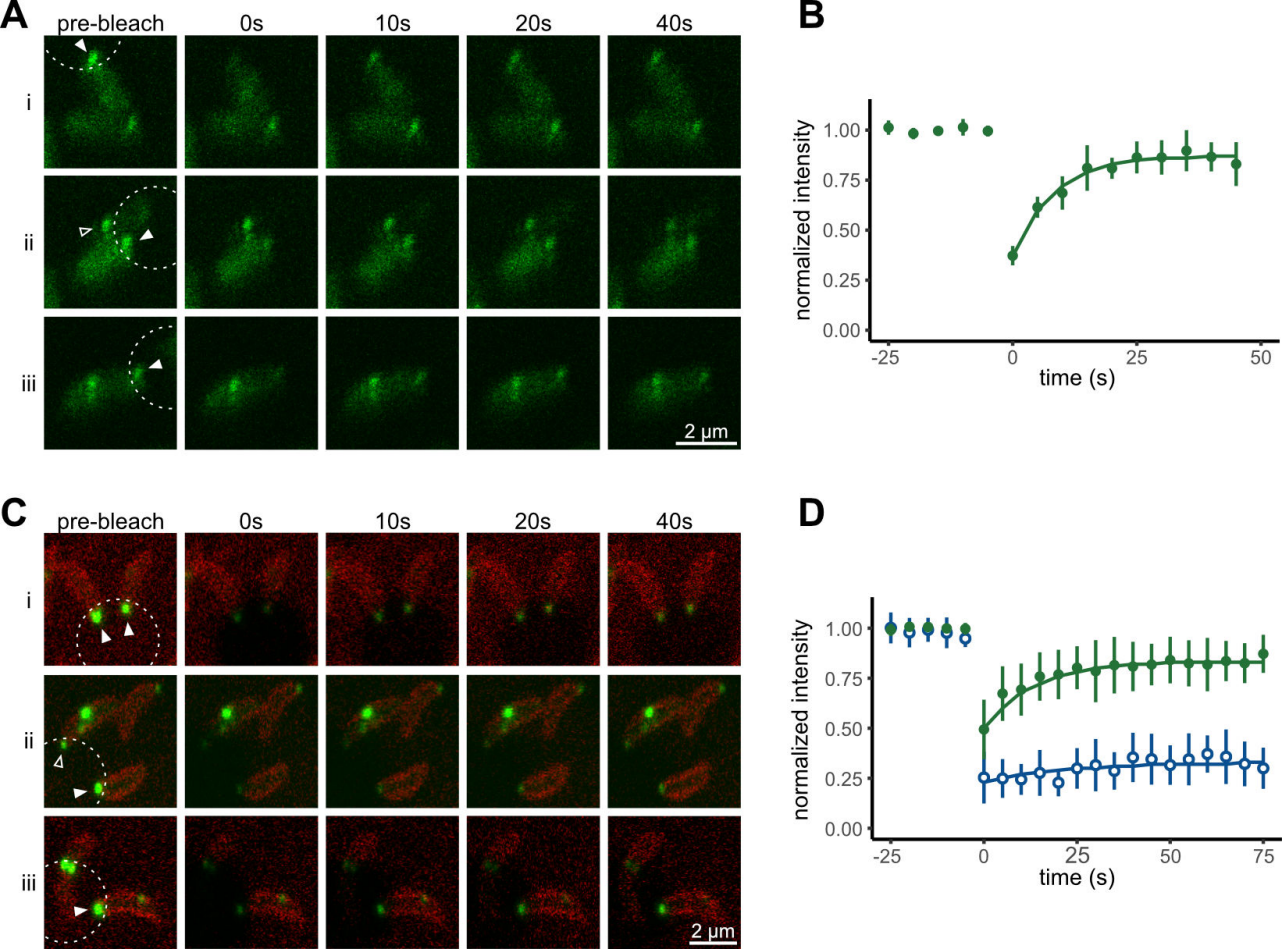
Polar FtsZ foci exhibit dynamic subunit exchange. (**A**) Time points taken from FRAP analyses of *A. tumefaciens* strain WM7687 expressing FtsZ_At_-mChart. The dotted circle indicates the region photobleached. Filled arrowheads indicate FtsZ_At_-mChart foci that recovered after photobleaching. The hollow arrowhead indicates a polar focus that lost fluorescence after bleaching of the remainder of the cell area. (**B**) Quantitation of focus intensity indicating mean (circle) and standard deviation (bar) at each time point for data collected from five cells. Focus intensity was normalized by total cellular intensity at each time point. The solid line represents the best-fit single-exponential recovery curve. The half-life of the combined data were 5.8 ± 1.0 s with a mobile fraction of 80%. (**C**) As in panel **A**, except with *C. crescentus* strain EG444 expressing a chromosomal FtsZ_Cc_-YFP fusion. Hollow arrowhead indicates polar FtsZ_Cc_-YFP foci in a cell that exhibited substantial midcell FtsZ localization. (**D**) Quantitation of FtsZ_Cc_-YFP focus intensity, indicating mean and standard deviation at each time point for polar FtsZ. In cells without midcell FtsZ (green, *n* = 8), polar FtsZ foci recovered with a half-life of 8.6 ± 1.6 s and a mobile fraction of 67%. In cells with midcell FtsZ (blue, *n* = 13), the half-life of polar focus recovery was 15.0 ± 9.7 s with a mobile fraction of 13%. Half-lives are reported as mean ± SE.

As an alternative approach, we tested the ability of polar FtsZ_At_-mChart foci in *A. tumefaciens* to dynamically exchange subunits, using FRAP. After selectively subjecting polar FtsZ_At_-mChart foci to photobleaching, foci exhibited significant fluorescence recovery within 10–20 s (Fig. 6A). Averaged FRAP recovery curves collected from several assays revealed that polar FtsZ_At_-mChart foci in *A. tumefaciens* recovered with a mean half-life of 5.8 ± 1.1 (SE) s (Fig. 6B), indicating rapid subunit turnover consistent with the earlier *in vitro* two-color capture assay (Fig. 2). Conversely, photobleaching a cell entirely except for the polar foci resulted in a gradual loss of fluorescence intensity of the foci (Fig. 6A, hollow arrowhead). Together, these results demonstrate that FtsZ_At_-mChart in polar foci actively exchanges with free protein in the cytoplasm, consistent with properties of condensates.

### *C. crescentus* FtsZ exhibits similar condensate-like properties in cells

Finally, we explored whether FtsZs from other Alphaproteobacteria might exhibit behaviors similar to FtsZ_At_. For this, we turned to *C. crescentus*, a well-characterized member of the Alphaproteobacteria order Caulobacterales, to contrast with *A. tumefaciens* from the order Hyphomicrobiales (syn. Rhizobiales). As mentioned above, *C. crescentus* FtsZ (FtsZ_Cc_) harbors an extended IDR that makes it a good candidate for condensate formation. As in *A. tumefaciens*, FtsZ in *C. crescentus* localizes to cell poles in newly divided cells, albeit for a shorter duration of the cell cycle (19, 20, 40).

When polar FtsZ_Cc_-YFP foci in *C. crescentus* cells were subjected to FRAP analysis, FtsZ_Cc_ foci recovered fluorescence much like FtsZ_At_ had in *A. tumefaciens* (Fig. 6C), yielding a mean half-life of 8.6 ± 1.6 (SE) s (Fig. 6D, green line). Interestingly, the recovery of polar FtsZ_Cc_-YFP foci was much slower in the subset of cells also containing midcell FtsZ bands or foci; in such cells, the mean half-life was 15.0 ± 9.7 (SE) s (Fig. 6D, blue line), likely reflecting differences in FtsZ regulation as the cell cycle progresses in each species. Despite this difference, polar FtsZ foci of both Alphaproteobacteria recovered fluorescence from a cytoplasmic pool of protein, consistent with a dynamic equilibrium of protein that would be expected of condensate formation in both species.

## DISCUSSION

To explore the potential formation of FtsZ condensates in *A. tumefaciens* and other Alphaproteobacteria, we investigated various *in vitro* and *in vivo* behaviors of FtsZ_At_. Purified FtsZ_At_ alone formed condensates under molecular crowding conditions, exhibited rapid equilibrium with the soluble protein fraction, and supported FtsZ polymerization in the presence of GTP. Examination of polar FtsZ_At_ foci in *A. tumefaciens* showed that these foci were also in equilibrium with a cytoplasmic pool of soluble protein and capable of coalescing. Furthermore, FtsZ_At_ expressed ectopically in *E. coli* formed similarly dynamic foci that interconverted into cytomotive FtsZ filaments in response to changes in the extracellular environment. Altogether, these data provide strong evidence that FtsZ_At_ forms biomolecular condensates through phase separation under a broad range of conditions *in vitro* with crowders, and *in vivo* as part of the typical *A. tumefaciens* cell cycle.

The shift between FtsZ_At_ foci and filaments in *E. coli* in response to changes in the extracellular environment suggests that assembly of FtsZ_At_ condensates is sensitive to the concentrations of some solutes, despite reduced sensitivity to high KCl levels compared to FtsZ_Ec_ condensates. Mounting cells on agarose pads may facilitate the observed shift toward FtsZ filament formation (Fig. S4A) through a mechanically stimulated calcium influx effect previously reported for *E. coli* visualized on agarose pads (41). Since increased calcium levels promote FtsZ bundling and polymerization *in vitro* (42), a similar effect might occur *in vivo* in this case. In contrast, cells placed in pure water would experience hypoosmotic shock, resulting in the rapid loss of cytoplasmic solutes such as glutamate and potassium ions (43). Like calcium, glutamate also promotes FtsZ polymerization (44); hence, a reduction in glutamate levels could help shift toward a higher pool of free FtsZ monomers. Likewise, as high potassium levels reduce condensate formation (Fig. S3B), reducing cellular potassium by hypoosmotic shock may promote condensation.

Like FtsZ_Ec_, FtsZ_At_ may interact with additional proteins to control condensate formation, especially since ectopic expression of FtsZ_At_ in *E. coli* required both overexpression and altered extracellular conditions to produce the FtsZ_At_-mChart foci that were found natively in *A. tumefaciens*. Although PopZ_At_ did not recruit FtsZ_At_, interactions with other proteins localized at the growth pole could drive FtsZ condensation. One possibility is the 2,115 amino acid GPR (growth pole ring) protein in *A. tumefaciens* (45, 46), and the analogous 882 amino acid TipN (tip of new pole) in the case of *C. crescentus* (47). Notably, both GPR and TipN feature disordered domains at their N and C termini, which might promote condensate formation, although neither condensates nor interactions with FtsZ have yet to be reported for either protein. Alternatively, a combination of intracellular conditions and extended linkers might promote FtsZ condensate formation throughout these cells, such that interaction with a negative regulator of phase separation at midcell could facilitate FtsZ polymerization where required. This mechanism would also be compatible with the non-polar FtsZ foci frequently observed in *A. tumefaciens* or *C. crescentus* prior to the formation of a midcell Z-ring (Fig. 1A and 6; Fig. S6A).

Currently, only one other FtsZ protein, FtsZ_Ec_ of *E. coli*, has been studied in the context of condensate formation. Robust biomolecular condensation of FtsZ_Ec_ *in vitro* is only observed under crowding cytomimetic conditions and in concert with partner regulatory proteins such as MatP or SlmA with its target DNA (11, 13). By itself, FtsZ_Ec_ only forms condensates at low KCl and high MgCl_2_ concentrations that strongly promote its oligomerization in the absence of GTP (12, 15). This suggests that although FtsZ_Ec_ may phase separate under certain conditions, additional heterotypic interactions are necessary to achieve sufficient multivalency for strong biomolecular condensation *in vitro*. In contrast, formation of FtsZ_At_ condensates required far lower concentrations of MgCl_2_ (1 mM, versus 10 mM for FtsZ_Ec_) and less molecular crowding. Moreover, FtsZ_At_ exhibited significantly decreased sensitivity to high salt concentrations compared with FtsZ_Ec_ (12), suggesting that the physical parameters for their condensate formation are different.

The core globular domains of both proteins are conserved, with 68% similar amino acid composition, whereas the FtsZ_At_ linker is nearly five times longer, suggesting that differences in condensate formation result primarily from the much longer linker domain of FtsZ_At_. The presence of this long IDR could increase hydrophobic interactions between FtsZ_At_ proteins, enhancing multivalency and decreasing sensitivity to high salt conditions compared to FtsZ_Ec_. The reduced formation of foci *in vivo* observed with the FtsZ_At_(Δ364-548) linker truncation further supports the idea that extended FtsZ linkers can play an important role in facilitating condensate formation. As linkers 3–6 times longer than in FtsZ_Ec_ are present in the vast majority of Alphaproteobacterial FtsZ proteins, the formation of FtsZ condensates may be a widely conserved feature across many Alphaproteobacterial species. FRAP analysis of polar FtsZ_Cc_ in *C. crescentus* demonstrated dynamic exchange between polar and cytoplasmic proteins (Fig. 6D), consistent with possible condensate formation in this organism. However, as formation of polar foci coincides with the lowest cellular FtsZ_Cc_ levels during the *C. crescentus* cell cycle (19, 48), whether these foci exist as condensates remains unclear. Future research will be required to explore how extended FtsZ linkers impact polar FtsZ_Cc_ foci and other potential FtsZ condensates across the diverse class of Alphaproteobacteria.

In sum, our results suggest that FtsZ condensates at the cell poles of Alphaproteobacteria such as *A. tumefaciens* and potentially *C. crescentus* may form to routinely sequester FtsZ pools during the cell cycle in between periods of active cell division. In contrast to bacteria such as *E. coli* that symmetrically expand via sidewall growth, have symmetrical cell poles, and are always poised to assemble the next Z-ring at midcell, *A. tumefaciens* cells grow at one pole for a period of time, followed by a transition to midcell division and then back to unipolar growth as part of their cell division cycle (23, 33, 34). *C. crescentus* grows through sidewall lengthening like *E. coli*, but like *A. tumefaciens*, each cell pole of *C. crescentus* has a distinct fate (swarmer vs. stalked) that differentially localizes multiple proteins to a specific pole, including a focus of FtsZ at the non-flagellated pole in nondividing cells (20). We suggest that unlike the obligatory FtsZ condensates formed in *A. tumefaciens* and potentially *C. crescentus*, *E. coli* cells might form FtsZ condensates only under more limited conditions, such as starvation or quiescence, that may benefit from the temporary sequestration of key cell division proteins. Such condensate formation in *E. coli* might be more highly regulated by partner proteins such as MatP or SlmA and their DNA target sequences to inhibit FtsZ phase separation except under these limited conditions.

## MATERIALS AND METHODS

### Strains, plasmids, and growth conditions

All strains and plasmids used for this study are listed in Table S1. *E. coli* and *A. tumefaciens* were cultured in Lennox LB media, and *C. crescentus* was cultured in PYE media. Unless otherwise indicated, *E. coli* cultures were grown at 37°C, *C. crescentus* at 30°C, and *A. tumefaciens* at 28°C. Liquid cultures were grown with shaking at 220 rpm. Strains containing plasmids were supplemented as needed with kanamycin (50 μg/mL) or chloramphenicol (15 μg/mL). Optical density of liquid cultures was assessed at 600 nm (OD_600_).

### Plasmid construction

Plasmid pWM7469 was generated using *in vivo E. coli* cloning (49). Vector DNA was amplified from plasmid pHYRSF53 using primers 2826 and 2827, and FtsZ_At_ was amplified from plasmid pJZ207 using primers 2828 and 2829. Each DNA fragment was transformed into *E. coli* strain SN1187 and grown with selection. Plasmid pWM7685 was generated by amplifying pJZ207 with primers 2979 and 2980, and amplifying mChartreuse from pNF02-mChartreuse using primers 2985 and 2986. The resulting fragments were digested with SacI and HindIII and ligated. Plasmid pWM7690 was generated by first amplifying mLychee from pNF02-mLychee using primers 3012 and 3013, then digesting the resulting product and pWM2633 with PstI and SpeI, followed by ligation. Plasmid pWM7691 was generated by amplifying *popZ* from *A. tumefaciens* C58 genomic DNA using primers 3014 and 3015, then digesting the product and pWM7690 with KpnI and BamHI, followed by ligation. Plasmid pWM7714 was generated by amplifying pWM7685 with primers 3098 and 3099, then ligating the resulting product to reform a circular plasmid. All constructs were verified by sequencing before use.

### Protein purification

Untagged FtsZ_At_ was purified using a two-step purification strategy to first isolate His-SUMO-FtsZ_At_, followed by scarless cleavage and removal of the N-terminal His-SUMO fragment and further purification by ion exchange chromatography. Briefly, His-SUMO-FtsZ_At_ was overproduced in *E. coli* C43 cells containing plasmid pMW7469, grown in LB Lennox broth with 50 µg/mL kanamycin, inducing with 1 mM IPTG at 37°C for 4 h. Cells were harvested by centrifugation and stored at −80°C. The pellet was thawed, resuspended in buffer A1 (50 mM Tris-HCl [pH 8.0], 500 mM KCl, and 10% glycerol) with 20 mM imidazole and supplemented with a free-EDTA tablet (Merck), 0.5 g/L lysozyme, 0.5 mM DTT, and 2 mM PMSF, and incubated for 15 min. After sonication and centrifugation, the soluble fraction was loaded onto a 5 mL HisTrap FF column (Cytiva) equilibrated in buffer A1 with 20 mM imidazole, and the protein was eluted with a linear gradient of imidazole (20 mM–1 M). Protein fractions were pooled, mixed with SUMO protease (1:20 protease:protein molar ratio), and dialyzed in 50 mM Tris-HCl, pH 8.0, 100 mM KCl, 10 mM imidazole, 0.1 mM EDTA, 10% glycerol, and 0.5 mM DTT. The untagged protein was further purified using a Ni-NTA His-bind resin (Merck) equilibrated in buffer A2 (50 mM Tris-HCl, pH 8.0, 0.1 mM EDTA, and 10% glycerol) with 100 mM KCl and 20 mM imidazole and a HiTrap Q HP column equilibrated in buffer A2 with 100 mM KCl. The protein was eluted with a 10-65% linear gradient of 1 M KCl in the same buffer. Protein purity was confirmed using SDS-PAGE. Fractions with pure protein were pooled and stored in aliquots at −80°C.

Protein concentration was assessed by Bradford (Bio-Rad Protein Assay) and UV spectrophotometry. GTPase activity was determined by quantification of the inorganic phosphate released using BIOMOL GREEN (Enzo Life Sciences) assay as previously described (50), at 5 µM FtsZ_At_. Reported range corresponds to values obtained for four independent protein purifications measured in duplicates (2.7 ± 0.2, 5.0 ± 0.2, 3.2 ± 0.1, and 3.6 ± 0.2 mol Pi · (mol FtsZ)^−1^ · min^−1^, values are mean ± SD). FtsZ depolymerization with time and the critical concentration of polymerization were assessed by measuring absorbance at 320 nm using a Cary 60 UV-vis spectrophotometer (Agilent Technologies) and a quartz cuvette (Hellma).

### Fluorescence labeling

FtsZ_At_ was covalently labeled with Alexa Fluor 488 or Alexa Fluor 647 carboxylic acid succinimidyl ester dyes (Thermo Fisher Scientific) at amino groups using a protocol in which the protein was assembled by CaCl_2_ and GTP, as described elsewhere (51), or an alternative protocol, with no difference observed in the behavior of the labeled protein in the experiments subsequently conducted. In the alternative protocol, FtsZ_At_ in 20 mM HEPES (pH 7.5), 50 mM KCl, 5 mM MgCl_2_, 2 mM EDTA was incubated with the dye (1:3 or 1:5 molar ratio for Alexa Fluor 488 or Alexa Fluor 647, respectively) for 30 min at room temperature, and free dye was removed by ultrafiltration using Vivaspin concentrators in buffer containing 50 mM Tris-HCl (pH 7.5), 300 mM KCl, 1 mM MgCl_2_. The labeling ratio, calculated from the molar absorption coefficients of the protein and the dyes (supplied by the manufacturer), was always below 1 mol dye/mol protein, with both protocols. The labeled protein was stored in aliquots at −80°C until use.

### Confocal microscopy

Samples were visualized in a Leica TCS SP5 inverted confocal microscope with a HCX PL APO 63× oil immersion objective (N.A. = 1.4; Leica, Mannheim, Germany) using 488 nm and 633 nm laser lines for excitation of Alexa Fluor 488 or Alexa Fluor 647, respectively, or with transmission light (DIC) for unlabeled samples, as described elsewhere (12). Solutions contained FtsZ_At_ with FtsZ_At_-Alexa 488 or FtsZ_At_-Alexa 647 as tracers and a crowder agent, Ficoll 70 (GE Healthcare) or dextran 500 (Sigma), previously dialyzed in 50 mM Tris-HCl, pH 7.5. The final buffer conditions were adjusted to be 50 mM Tris-HCl, pH 7.5, 1 mM MgCl_2_, 100 mM KCl, unless otherwise specified. Samples were incubated for 30 min and visualized using silicone chambers glued to coverslips. Several images corresponding to different fields were acquired. For capture experiments, fresh FtsZ_At_-Alexa 488 was added to condensates containing FtsZ_At_ and FtsZ_At_-Alexa 647 as tracer, gently mixed, and imaged with time. Images shown are representative of three independent experiments, prepared using ImageJ (52), which was also used to obtain the intensity profiles. Only when specified, brightness was increased using Microsoft PowerPoint (correction applied uniformly to the whole image). In grouped images, the scale bar length is only indicated in one of the images.

### Turbidity measurements

Absorbance determinations were conducted in a Varioskan Flash Plate reader (Thermo Fisher Scientific, MA, USA), as previously described (13). In brief, 85 μL samples, equivalent to those prepared for confocal imaging but without fluorescently labeled protein, were loaded in 384-well clear polystyrene flat-bottom microplates (Greiner Bio-One) and incubated for 30 min at room temperature prior to measuring absorbance at 350 nm. Reported data are the mean of three independent experiments ±SD. C_sat_ for condensate formation was determined by fitting a linear model to the data with user-written scripts and functions in MATLAB (ver. 7.10; MathWorks, Natick, MA) as described (13), and error was obtained by propagation from the errors of the slope and the y-intercept, representing confidence limits at 68%.

### Transmission electron microscopy

Transmission electron microscopy experiments were conducted using a Thermo Fisher TALOS L120C transmission electron microscope operated at 120 kV and with a Thermo Fisher CETA-F camera. Images were recorded under low-dose conditions. Unless otherwise stated, after incubation with 2 mM GTP for 5 min, FtsZ_At_ samples were adsorbed to glow-discharged carbon-coated grids and stained with 2% uranyl acetate.

### Widefield fluorescence microscopy

For routine imaging of *A. tumefaciens*, overnight cultures were diluted to an OD_600_ of 0.1 in LB and incubated for 2 h. IPTG was added to 100 μM, and cells were incubated for an additional 1.5 h. Otherwise, cultures were induced as described. After the induction period, an aliquot of the culture was centrifuged and concentrated to an OD_600_ of ~4.0. Cells were mounted on a 2% agarose pad prepared with M9 media supplemented with 0.2% glucose, 0.2% acid casein peptone, and 10 μg/mL thiamine. For imaging *E. coli*, overnight cultures were diluted 1:200 in LB and grown for 2 h prior to induction as described. When applicable, Hoechst 33342 and FM4-64 were added to a concentration of 2 μM for the final 30 or 15 min of culture growth, respectively. Widefield imaging was conducted with an Olympus BX63 microscope equipped with a Hamamatsu C11440 ORCA-Spark digital CMOS camera using cellSens software (Olympus).

### FRAP

*A. tumefaciens* cultures were grown as described above, except that inductions were for 2 h with 1 mM IPTG. For *C. crescentus,* cultures of EG444 were diluted in PYE media to an OD_600_ of 0.1 and allowed to grow for 3 h prior to induction with 0.3% xylose for 1 h. *C. crescentus* was mounted on 2% agarose pads prepared with 2 μM FM4-64 in a modified PYE media (0.5 mM CaCl_2_, 1 mM MgSO_4_, 0.2% glucose, and 0.2% acid casein peptone). FRAP image acquisition was performed with a Nikon A1R confocal laser microscope at the Center for Advanced Microscopy in the Department of Integrative Biology & Pharmacology at McGovern Medical School, UTHealth Houston.

### Image analysis

Basic image analysis was performed using Fiji/ImageJ. For generating demographs, cell segmentation was first performed using Cellpose-SAM (53), then fluorescence profiles collected and visualized with MicrobeJ (54), using FM4-64 staining intensity to identify growth poles. Initial FRAP analysis was performed using EasyFRAP-web (55), and the results were subsequently visualized using R (56) and ggplot2 (57). The standard error of half-life estimates was calculated using the delta standard error method.

## Data Availability

All study data discussed in the paper are available in the main text and supplemental material.

## SUPPLEMENTAL MATERIAL

Supplemental material mbio.00494-26-s0001.pdf Supplemental figures and tables.

Movie S1 mbio.00494-26-s0002.mp4

Movie S2 mbio.00494-26-s0003.mp4

Movie S3 mbio.00494-26-s0004.mp4

ASM does not own the copyrights to Supplemental Material that may be linked to, or accessed through, an article. The authors have granted ASM a non-exclusive, world-wide license to publish the Supplemental Material files. Please contact the corresponding author directly for reuse.

## References

[1] Banani SF, Lee HO, Hyman AA, Rosen MK. 2017. Biomolecular condensates: organizers of cellular biochemistry. Nat Rev Mol Cell Biol 18:285–298. doi:10.1038/nrm.2017.7 28225081 PMC7434221

[2] Shin Y, Brangwynne CP. 2017. Liquid phase condensation in cell physiology and disease. Science 357:eaaf4382. doi:10.1126/science.aaf4382 28935776

[3] Alberti S, Dormann D. 2019. Liquid-liquid phase separation in disease. Annu Rev Genet 53:171–194. doi:10.1146/annurev-genet-112618-043527 31430179

[4] Alberti S, Hyman AA. 2021. Biomolecular condensates at the nexus of cellular stress, protein aggregation disease and ageing. Nat Rev Mol Cell Biol 22:196–213. doi:10.1038/s41580-020-00326-6 33510441

[5] Azaldegui CA, Vecchiarelli AG, Biteen JS. 2021. The emergence of phase separation as an organizing principle in bacteria. Biophys J 120:1123–1138. doi:10.1016/j.bpj.2020.09.023 33186556 PMC8059088

[6] Monterroso B, Margolin W, Boersma AJ, Rivas G, Poolman B, Zorrilla S. 2024. Macromolecular crowding, phase separation, and homeostasis in the orchestration of bacterial cellular functions. Chem Rev 124:1899–1949. doi:10.1021/acs.chemrev.3c00622 38331392 PMC10906006

[7] Barrows JM, Goley ED. 2021. FtsZ dynamics in bacterial division: what, how, and why? Curr Opin Cell Biol 68:163–172. doi:10.1016/j.ceb.2020.10.013 33220539 PMC7925355

[8] Cameron TA, Margolin W. 2024. Insights into the assembly and regulation of the bacterial divisome. Nat Rev Microbiol 22:33–45. doi:10.1038/s41579-023-00942-x 37524757 PMC11102604

[9] Ramm B, Schumacher D, Harms A, Heermann T, Klos P, Müller F, Schwille P, Søgaard-Andersen L. 2023. Biomolecular condensate drives polymerization and bundling of the bacterial tubulin FtsZ to regulate cell division. Nat Commun 14:3825. doi:10.1038/s41467-023-39513-2 37380708 PMC10307791

[10] Xu P, Schumacher D, Liu C, Harms A, Dickmanns M, Beck F, Plitzko JM, Baumeister W, Søgaard-Andersen L. 2025. In situ architecture of a nucleoid-associated biomolecular co-condensate that regulates bacterial cell division. Proc Natl Acad Sci USA 122:e2419610121. doi:10.1073/pnas.2419610121 39739804 PMC11725790

[11] Monterroso B, Zorrilla S, Sobrinos‐Sanguino M, Robles‐Ramos MA, López‐Álvarez M, Margolin W, Keating CD, Rivas G. 2019. Bacterial FtsZ protein forms phase‐separated condensates with its nucleoid‐associated inhibitor SlmA. EMBO Rep 20. doi:10.15252/embr.201845946 PMC6322363 30523075

[12] Robles-Ramos MÁ, Zorrilla S, Alfonso C, Margolin W, Rivas G, Monterroso B. 2021. Assembly of bacterial cell division protein FtsZ into dynamic biomolecular condensates. Biochim Biophys Acta Mol Cell Res 1868:118986. doi:10.1016/j.bbamcr.2021.118986 33581219 PMC8529516

[13] Barros-Medina I, Robles-Ramos MÁ, Sobrinos-Sanguino M, Luque-Ortega JR, Alfonso C, Margolin W, Rivas G, Monterroso B, Zorrilla S. 2025. Evidence for biomolecular condensates formed by the Escherichia coli MatP protein in spatiotemporal regulation of the bacterial cell division cycle. Int J Biol Macromol 309:142691. doi:10.1016/j.ijbiomac.2025.142691 40174834 PMC12756933

[14] Yu J, Liu Y, Yin H, Chang Z. 2019. Regrowth-delay body as a bacterial subcellular structure marking multidrug-tolerant persisters. Cell Discov 5:8. doi:10.1038/s41421-019-0080-3 30675381 PMC6341109

[15] Rivas G, López A, Mingorance J, Ferrándiz MJ, Zorrilla S, Minton AP, Vicente M, Andreu JM. 2000. Magnesium-induced linear self-association of the FtsZ bacterial cell division protein monomer. J Biol Chem 275:11740–11749. doi:10.1074/jbc.275.16.11740 10766796

[16] Guan F, Yu J, Yu J, Liu Y, Li Y, Feng X-H, Huang KC, Chang Z, Ye S. 2018. Lateral interactions between protofilaments of the bacterial tubulin homolog FtsZ are essential for cell division. eLife 7:e35578. doi:10.7554/eLife.35578 29889022 PMC6050046

[17] Vaughan S, Wickstead B, Gull K, Addinall SG. 2004. Molecular evolution of FtsZ protein sequences encoded within the genomes of archaea, bacteria, and eukaryota. J Mol Evol 58:19–29. doi:10.1007/s00239-003-2523-5 14743312

[18] Buske PJ, Levin PA. 2013. A flexible C-terminal linker is required for proper FtsZ assembly in vitro and cytokinetic ring formation in vivo. Mol Microbiol 89:249–263. doi:10.1111/mmi.12272 23692518 PMC3708608

[19] Quardokus EM, Din N, Brun YV. 2001. Cell cycle and positional constraints on FtsZ localization and the initiation of cell division in Caulobacter crescentus. Mol Microbiol 39:949–959. doi:10.1046/j.1365-2958.2001.02287.x 11251815

[20] Thanbichler M, Shapiro L. 2006. MipZ, a spatial regulator coordinating chromosome segregation with cell division in Caulobacter. Cell 126:147–162. doi:10.1016/j.cell.2006.05.038 16839883

[21] Zupan JR, Cameron TA, Anderson-Furgeson J, Zambryski PC. 2013. Dynamic FtsA and FtsZ localization and outer membrane alterations during polar growth and cell division in Agrobacterium tumefaciens. Proc Natl Acad Sci USA 110:9060–9065. doi:10.1073/pnas.1307241110 23674672 PMC3670362

[22] Cameron TA, Anderson-Furgeson J, Zupan JR, Zik JJ, Zambryski PC. 2014. Peptidoglycan synthesis machinery in Agrobacterium tumefaciens during unipolar growth and cell division. mBio 5:e01219-14. doi:10.1128/mBio.01219-14 24865559 PMC4045076

[23] Grangeon R, Zupan JR, Anderson-Furgeson J, Zambryski PC. 2015. PopZ identifies the new pole, and PodJ identifies the old pole during polar growth in Agrobacterium tumefaciens. Proc Natl Acad Sci USA 112:11666–11671. doi:10.1073/pnas.1515544112 26324921 PMC4577194

[24] Howell M, Aliashkevich A, Sundararajan K, Daniel JJ, Lariviere PJ, Goley ED, Cava F, Brown PJB. 2019. Agrobacterium tumefaciens divisome proteins regulate the transition from polar growth to cell division. Mol Microbiol 111:1074–1092. doi:10.1111/mmi.14212 30693575 PMC6482847

[25] de Boer P, Crossley R, Rothfield L. 1992. The essential bacterial cell-division protein FtsZ is a GTPase. Nature 359:254–256. doi:10.1038/359254a0 1528268

[26] Erickson HP, Taylor DW, Taylor KA, Bramhill D. 1996. Bacterial cell division protein FtsZ assembles into protofilament sheets and minirings, structural homologs of tubulin polymers. Proc Natl Acad Sci USA 93:519–523. doi:10.1073/pnas.93.1.519 8552673 PMC40269

[27] Mukherjee A, Lutkenhaus J. 1999. Analysis of FtsZ assembly by light scattering and determination of the role of divalent metal cations. J Bacteriol 181:823–832. doi:10.1128/JB.181.3.823-832.1999 9922245 PMC93448

[28] Woodruff JB, Ferreira Gomes B, Widlund PO, Mahamid J, Honigmann A, Hyman AA. 2017. The centrosome is a selective condensate that nucleates microtubules by concentrating tubulin. Cell 169:1066–1077. doi:10.1016/j.cell.2017.05.028 28575670

[29] Fraikin N, Couturier A, Mercier R, Lesterlin C. 2025. A palette of bright and photostable monomeric fluorescent proteins for bacterial time-lapse imaging. Sci Adv 11:eads6201. doi:10.1126/sciadv.ads6201 40238862 PMC12002091

[30] Ma X, Sun Q, Wang R, Singh G, Jonietz EL, Margolin W. 1997. Interactions between heterologous FtsA and FtsZ proteins at the FtsZ ring. J Bacteriol 179:6788–6797. doi:10.1128/jb.179.21.6788-6797.1997 9352931 PMC179610

[31] Lasker K, Boeynaems S, Lam V, Scholl D, Stainton E, Briner A, Jacquemyn M, Daelemans D, Deniz A, Villa E, Holehouse AS, Gitler AD, Shapiro L. 2022. The material properties of a bacterial-derived biomolecular condensate tune biological function in natural and synthetic systems. Nat Commun 13:5643. doi:10.1038/s41467-022-33221-z 36163138 PMC9512792

[32] Saurabh S, Chong TN, Bayas C, Dahlberg PD, Cartwright HN, Moerner WE, Shapiro L. 2022. ATP-responsive biomolecular condensates tune bacterial kinase signaling. Sci Adv 8:eabm6570. doi:10.1126/sciadv.abm6570 35171683 PMC8849385

[33] Grangeon R, Zupan J, Jeon Y, Zambryski PC. 2017. Loss of PopZ_At_ activity in Agrobacterium tumefaciens by deletion or depletion leads to multiple growth poles, minicells, and growth defects. mBio 8:e01881–17. doi: 10.1128/mBio.01881-17 29138309 10.1128/mBio.01881-17 PMC5686542

[34] Howell M, Aliashkevich A, Salisbury AK, Cava F, Bowman GR, Brown PJB. 2017. Absence of the polar organizing protein PopZ results in reduced and asymmetric cell division in Agrobacterium tumefaciens. J Bacteriol 199:e00101-17. doi:10.1128/JB.00101-17 28630123 PMC5553032

[35] Kroschwald S, Maharana S, Simon A. 2017. Hexanediol: a chemical probe to investigate the material properties of membrane-less compartments. Matters:1–7. doi:10.19185/matters.201702000010

[36] Düster R, Kaltheuner IH, Schmitz M, Geyer M. 2021. 1,6-hexanediol, commonly used to dissolve liquid-liquid phase separated condensates, directly impairs kinase and phosphatase activities. J Biol Chem 296:100260. doi:10.1016/j.jbc.2021.100260 33814344 PMC7948595

[37] Meduri R, Rubio LS, Mohajan S, Gross DS. 2022. Phase-separation antagonists potently inhibit transcription and broadly increase nucleosome density. J Biol Chem 298:102365. doi:10.1016/j.jbc.2022.102365 35963432 PMC9486037

[38] Meier EL, Razavi S, Inoue T, Goley ED. 2016. A novel membrane anchor for FtsZ is linked to cell wall hydrolysis in Caulobacter crescentus. Mol Microbiol 101:265–280. doi:10.1111/mmi.13388 27028265 PMC4935632

[39] Naha A, Haeusser DP, Margolin W. 2023. Anchors: a way for FtsZ filaments to stay membrane bound. Mol Microbiol 120:525–538. doi:10.1111/mmi.15067 37503768 PMC10593102

[40] Schofield WB, Lim HC, Jacobs-Wagner C. 2010. Cell cycle coordination and regulation of bacterial chromosome segregation dynamics by polarly localized proteins. EMBO J 29:3068–3081. doi:10.1038/emboj.2010.207 20802464 PMC2944072

[41] Bruni GN, Weekley RA, Dodd BJT, Kralj JM. 2017. Voltage-gated calcium flux mediates Escherichia coli mechanosensation. Proc Natl Acad Sci USA 114:9445–9450. doi:10.1073/pnas.1703084114 28808010 PMC5584419

[42] Yu X-C, Margolin W. 1997. Ca2+-mediated GTP-dependent dynamic assembly of bacterial cell division protein FtsZ into asters and polymer networks in vitro. EMBO J 16:5455–5463. doi:10.1093/emboj/16.17.5455 9312004 PMC1170176

[43] Schleyer M, Schmid R, Bakker EP. 1993. Transient, specific and extremely rapid release of osmolytes from growing cells of Escherichia coli K-12 exposed to hypoosmotic shock. Arch Microbiol 160:424–431. doi:10.1007/BF00245302 8297208

[44] Beuria TK, Krishnakumar SS, Sahar S, Singh N, Gupta K, Meshram M, Panda D. 2003. Glutamate-induced assembly of bacterial cell division protein FtsZ. J Biol Chem 278:3735–3741. doi:10.1074/jbc.M205760200 12446699

[45] Zupan JR, Grangeon R, Robalino-Espinosa JS, Garnica N, Zambryski P. 2019. Growth pole ring protein forms a 200-nm-diameter RING structure essential for polar growth and rod shape in Agrobacterium tumefaciens. Proc Natl Acad Sci USA 116:10962–10967. doi:10.1073/pnas.1905900116 31085632 PMC6561148

[46] Zupan J, Guo Z, Biddle T, Zambryski P. 2021. Agrobacterium tumefaciens growth pole ring protein: C terminus and internal apolipoprotein homologous domains are essential for function and subcellular localization. mBio 12:e00764–21. doi:10.1128/mBio.00764-21 34006657 PMC8262873

[47] Lam H, Schofield WB, Jacobs-Wagner C. 2006. A landmark protein essential for establishing and perpetuating the polarity of a bacterial cell. Cell 124:1011–1023. doi:10.1016/j.cell.2005.12.040 16530047

[48] Goley ED, Dye NA, Werner JN, Gitai Z, Shapiro L. 2010. Imaging-based identification of a critical regulator of FtsZ protofilament curvature in Caulobacter. Mol Cell 39:975–987. doi:10.1016/j.molcel.2010.08.027 20864042 PMC2945607

[49] Nozaki S, Niki H. 2019. Exonuclease III (XthA) enforces in vivo DNA cloning of Escherichia coli to create cohesive ends. J Bacteriol 201:e00660-18. doi:10.1128/JB.00660-18 30530516 PMC6379578

[50] Suigo L, Monterroso B, Sobrinos-Sanguino M, Alfonso C, Straniero V, Rivas G, Zorrilla S, Valoti E, Margolin W. 2023. Benzodioxane-benzamides as promising inhibitors of Escherichia coli FtsZ. Int J Biol Macromol 253:126398. doi:10.1016/j.ijbiomac.2023.126398 37634788

[51] González JM, Jiménez M, Vélez M, Mingorance J, Andreu JM, Vicente M, Rivas G. 2003. Essential cell division protein FtsZ assembles into one monomer-thick ribbons under conditions resembling the crowded intracellular environment. J Biol Chem 278:37664–37671. doi:10.1074/jbc.M305230200 12807907

[52] Schneider CA, Rasband WS, Eliceiri KW. 2012. NIH Image to ImageJ: 25 years of image analysis. Nat Methods 9:671–675. doi:10.1038/nmeth.2089 22930834 PMC5554542

[53] Pachitariu M, Rariden M, Stringer C. 2025. Cellpose-SAM: superhuman generalization for cellular segmentation. bioRxiv. doi:10.1101/2025.04.28.651001

[54] Ducret A, Quardokus EM, Brun YV. 2016. MicrobeJ, a tool for high throughput bacterial cell detection and quantitative analysis. Nat Microbiol 1:16077. doi:10.1038/nmicrobiol.2016.77 27572972 PMC5010025

[55] Koulouras G, Panagopoulos A, Rapsomaniki MA, Giakoumakis NN, Taraviras S, Lygerou Z. 2018. EasyFRAP-web: a web-based tool for the analysis of fluorescence recovery after photobleaching data. Nucleic Acids Res 46:W467–W472. doi:10.1093/nar/gky508 29901776 PMC6030846

[56] R Core Team. 2025. R: a language and environment for statistical computing. R Foundation for Statistical Computing, Vienna, Austria.

[57] Wickham H. 2016. Ggplot2: elegant graphics for data analysis. Springer Verlag, New York. https://ggplot2.tidyverse.org.

