# Supplementary material for: FtsZ forms biomolecular condensates in a polar-growing Alphaproteobacterium
Source: mBio. 2026 Apr 15;17(5):e00494-26. doi: 10.1128/mbio.00494-26 (PMC13170164; doi:10.1128/mbio.00494-26)
Supplement: Supplemental material — Supplemental figures and tables. [file mbio.00494-26-s0001.pdf]

FtsZ forms biomolecular condensates in a polar-growing Alphaproteobacterium

Todd A. Cameron[1], Miguel Ángel Robles-Ramos[2], Lorenzo Suigo[1], Silvia Zorrilla[2], William Margolin[1]

[1] University of Texas Health Science Center at Houston, Houston, TX.
[2] Centro de Investigaciones Biológicas Margarita Salas, Consejo Superior de Investigaciones Científicas (CSIC), Madrid, Spain.

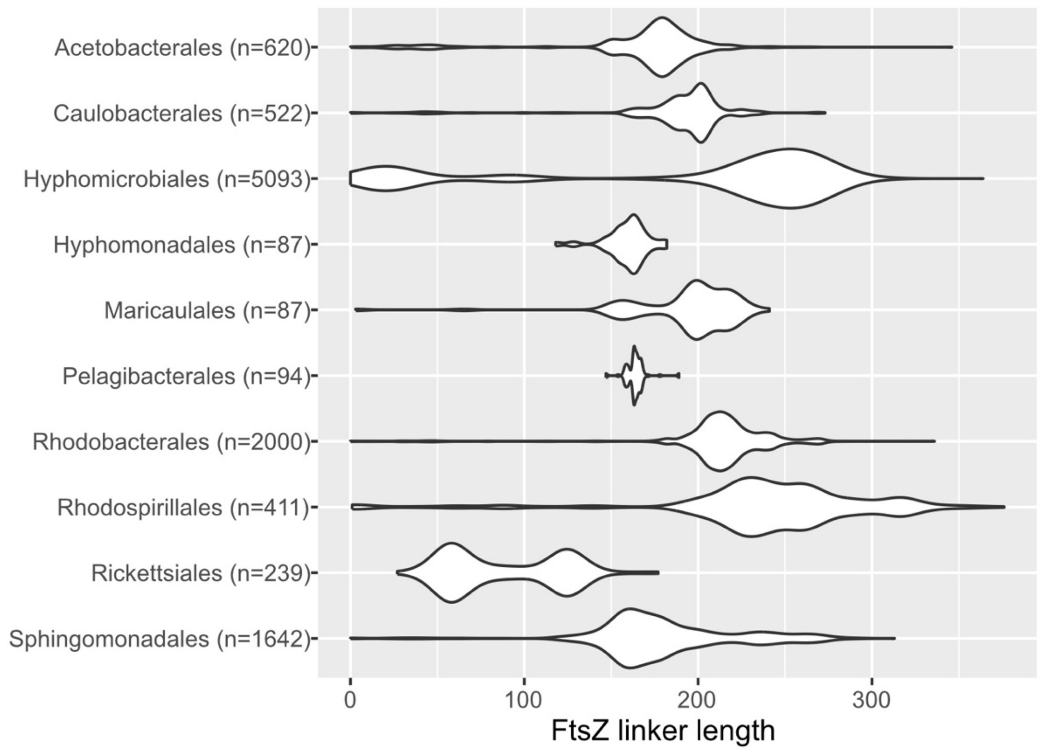

Fig S1 FtsZ linker lengths among major Alphaproteobacteria orders. Density plots of linker lengths for Alphaproteobacterial proteins identified as FtsZ in the NCBI RefSeq database with a minimum total length of 300 amino acids. Linker lengths were determined by measuring the number of residues between the FtsZ core domain and the conserved C-terminal peptide, if present. Sequences were aligned and domain boundaries determined by comparison to the *E. coli* MG1655 FtsZ protein. Aside from Rickettsiales, the majority of FtsZ linkers in every other order are three or more times longer than the 50 amino acid linker of the *E. coli* MG1655 FtsZ protein. The smaller peak of Hyphomicrobiales (syn. Rhizobiales) linkers less than 50 amino acids primarily originates from FtsZ proteins that lack identifiable C-terminal peptides and likely represent truncated duplications of FtsZ that are common among some members of this order.

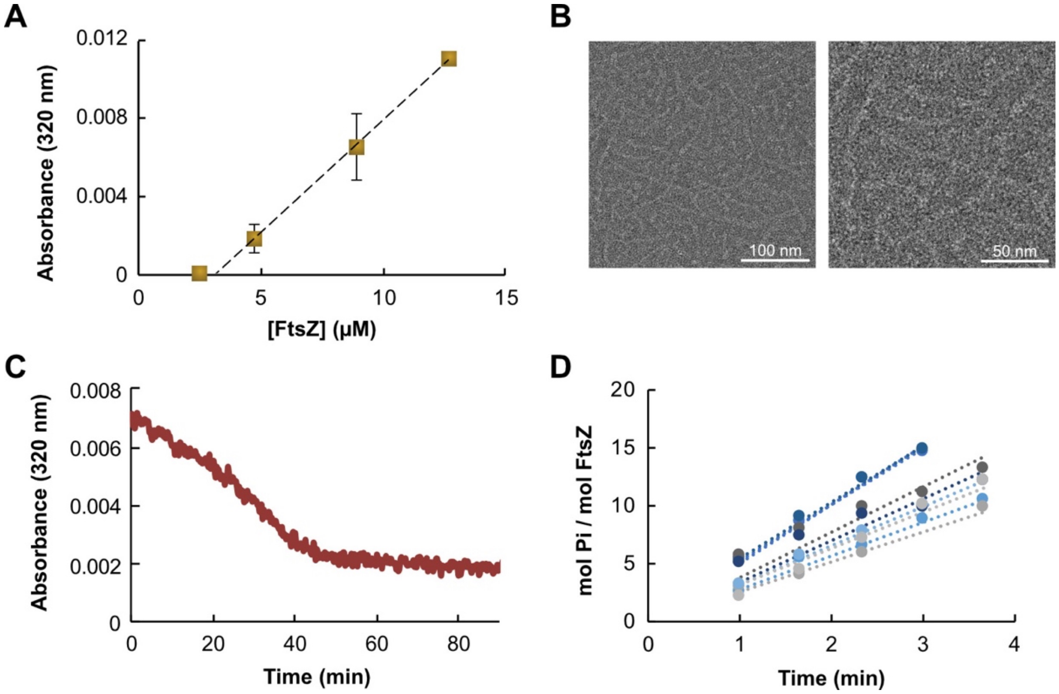

Fig S2 Properties of purified $FtsZ_{At}$. (**A**) FtsZ polymerization was assessed at different concentrations of protein by measuring absorbance at 320 nm. Linear regression of the data indicated a critical concentration of approximately 3 μM FtsZ. Polymerization was assessed in buffer containing 50 mM Tris-HCl pH 8.0, 300 mM KCl, 10% glycerol, 0.1 mM EDTA, 5 mM $MgCl_2$ and 2 mM GTP. Data correspond to the mean ± SD from two independent measurements, except for the point at higher concentration which was measured once. (**B**) Transmission electron microscopy of 7 μM FtsZ in buffer containing 50 mM Tris-HCl pH 7.5, 300 mM KCl, 1 mM $MgCl_2$, and 2 mM GTP. Sample staining was performed after a 5 min incubation period. Two images are shown, with different magnification. (**C**) Depolymerization of 8.9 μM FtsZ polymerized in buffer containing 50 mM Tris-HCl pH 7.5, 300 mM KCl, 5 mM $MgCl_2$, and 0.75 mM GTP. (**D**) GTPase activity of $FtsZ_{At}$ measured as mol Pi generated per mol of $FtsZ_{At}$ in the solution (5 μM) over time. Reactions were carried out using different stocks of purified FtsZ in the same buffer as FtsZ polymerization in (A).

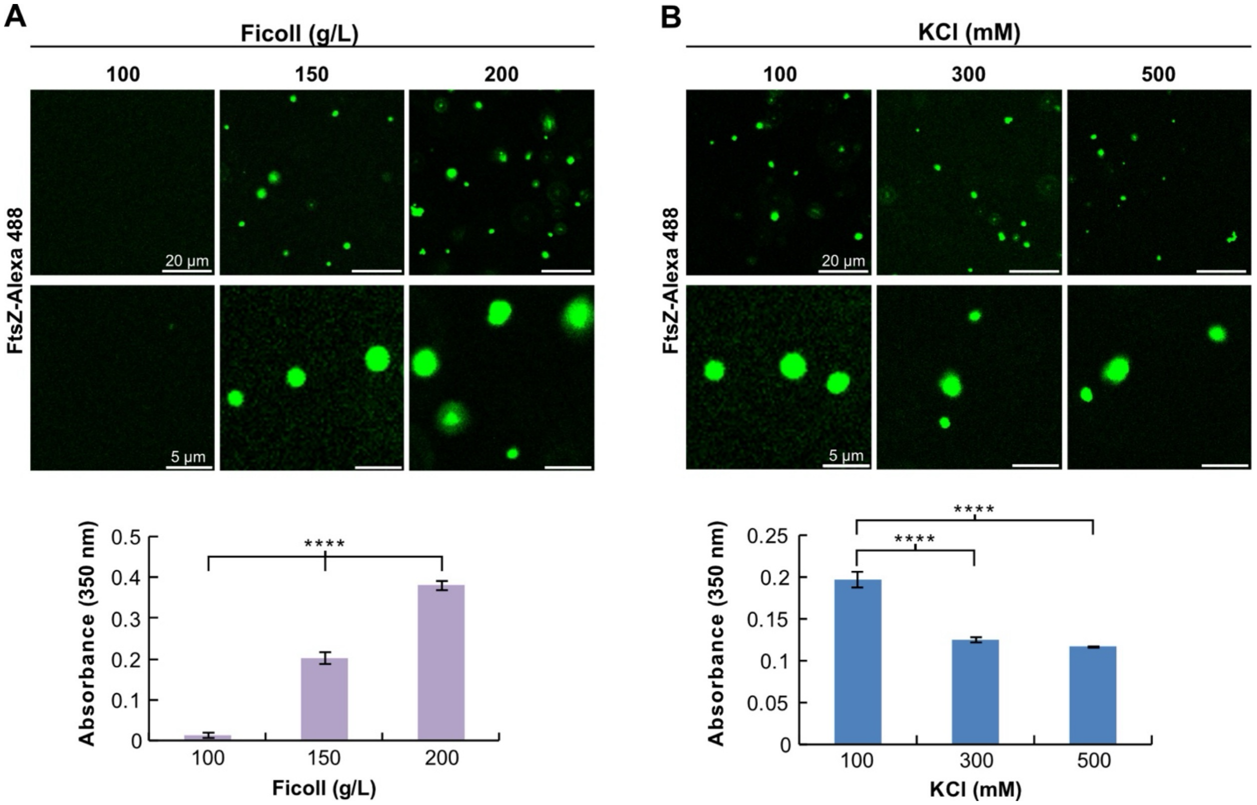

Fig S3 Crowding strongly promotes $FtsZ_{At}$ condensation whereas ionic strength has a minor impact. Confocal images (top) and turbidity measurements (bottom) of $FtsZ_{At}$ with varying (**A**) Ficoll or (**B**) KCl concentration. The lower row of confocal images shows a higher magnification of the same conditions as in the upper row. Samples of $FtsZ_{At}$ (7 μM), including $FtsZ_{At}$-Alexa 488 (0.5 μM) for fluorescence micrographs, were incubated for 30 min before measurement. Turbidity was assessed by absorbance at 350 nm of the same samples without the labeled protein. Statistical significance was assessed by one-way ANOVA followed by Tukey's test: **** $P < 0.0001$. Experiments were performed using Ficoll as the crowding agent at (A) the specified concentration or (B) 150 g/L, in 50 mM Tris-HCl, pH 7.5, and 1 mM $MgCl_2$ supplemented with KCl at (A) 100 mM or (B) the indicated concentration.

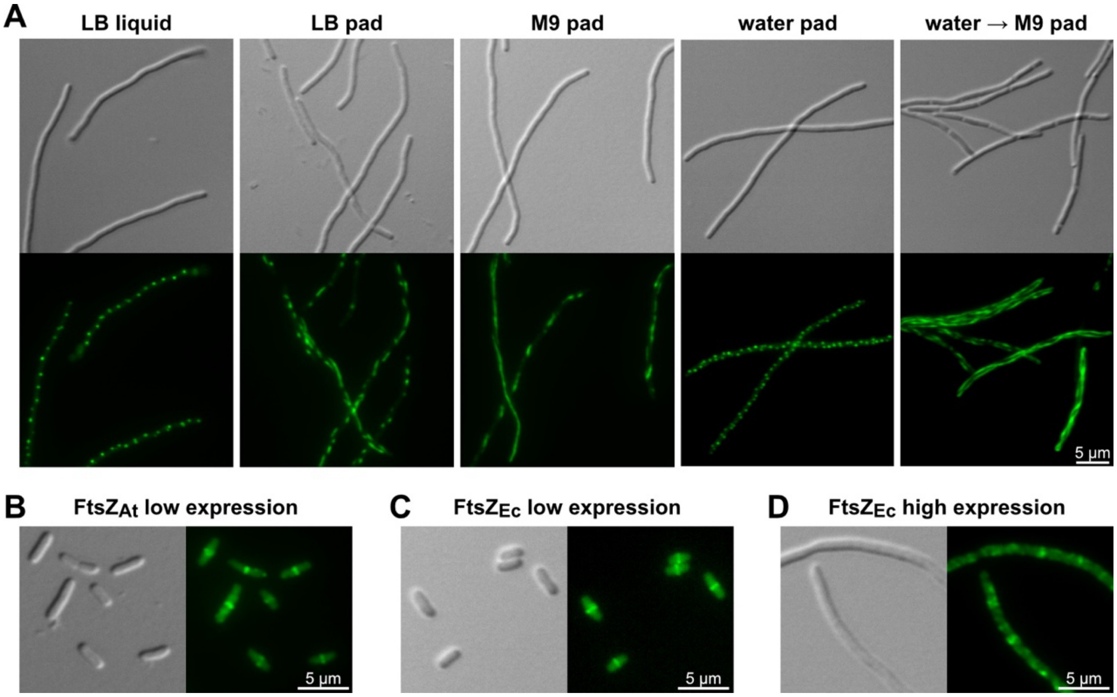

Fig S4 $FtsZ_{At}$ foci and filaments under different conditions in *E. coli.* (**A**) Localization of $FtsZ_{At}$-mChart expressed at high levels in *E. coli* strain WM7689 with 100 μM IPTG was affected by shifts to different media and buffer conditions. A single culture was grown to early stationary phase and aliquots were washed and mounted on agarose pads with the indicated media or water. Cells in the original LB culture presented FtsZ foci, whereas cells mounted on LB agarose pads or M9-glucose agarose pads developed long FtsZ filaments instead. Cells washed with water formed FtsZ foci on water agarose pads, or filaments when mounted on M9-glucose agarose pads. (**B**) $FtsZ_{At}$-mChart expressed in *E. coli* strain WM7689 with 50 μM IPTG for 2 h localized in canonical ring-like patterns. (**C**) *E. coli* FtsZ-GFP expressed in *E. coli* strain WM2754 from a sodium salicylate inducible plasmid localizes as rings without induction, or (**D**) as a mix of rings, patches, and non-discrete patterns when overexpressed with 2 μM sodium salicylate for 1 h.

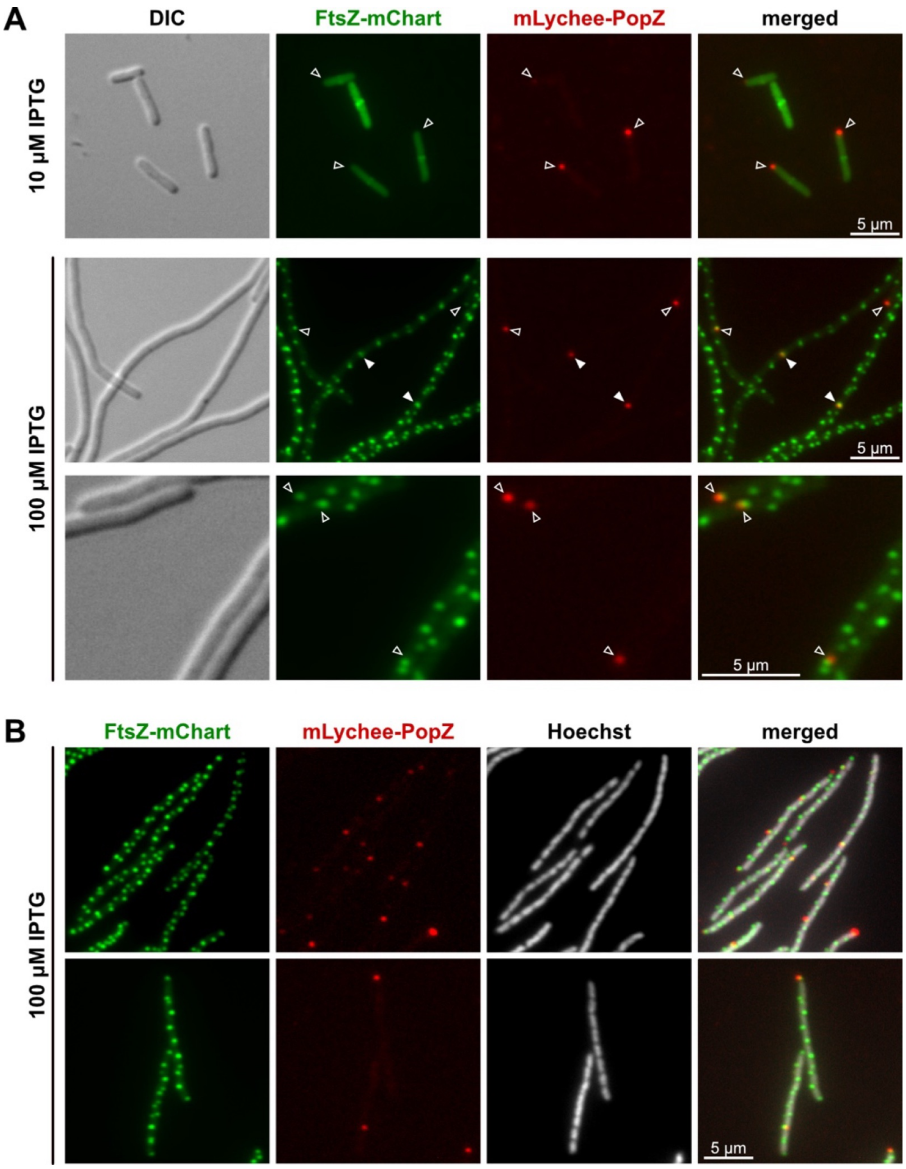

Fig S5 $FtsZ_{At}$-mChart and mLychee-$PopZ_{At}$ foci co-localize to nucleoid-free regions in *E. coli.* (**A**) $FtsZ_{At}$-mChart (green) was co-expressed with mLychee-$PopZ_{At}$ (red) in *E. coli* strain WM7692 at low (10 µM IPTG) or high (100 µM IPTG) FtsZ expression levels for 2 h and imaged on water-agarose pads. Expression of mLychee-PopZ was induced by 10 µM sodium salicylate for 3 h. When FtsZ-mChart was expressed at low levels, it did not colocalize with mLychee-PopZ (hollow arrowheads). However, at higher expression levels of FtsZ-mChart, PopZ foci were nearly universally either closely associated with or colocalized with an FtsZ focus (filled arrowheads). (**B**) When cells induced with 100 µM IPTG were stained with Hoechst 33342 to visualize nucleoids (grey), foci of both proteins primarily localized between nucleoids and in the nucleoid-free regions of cell poles.

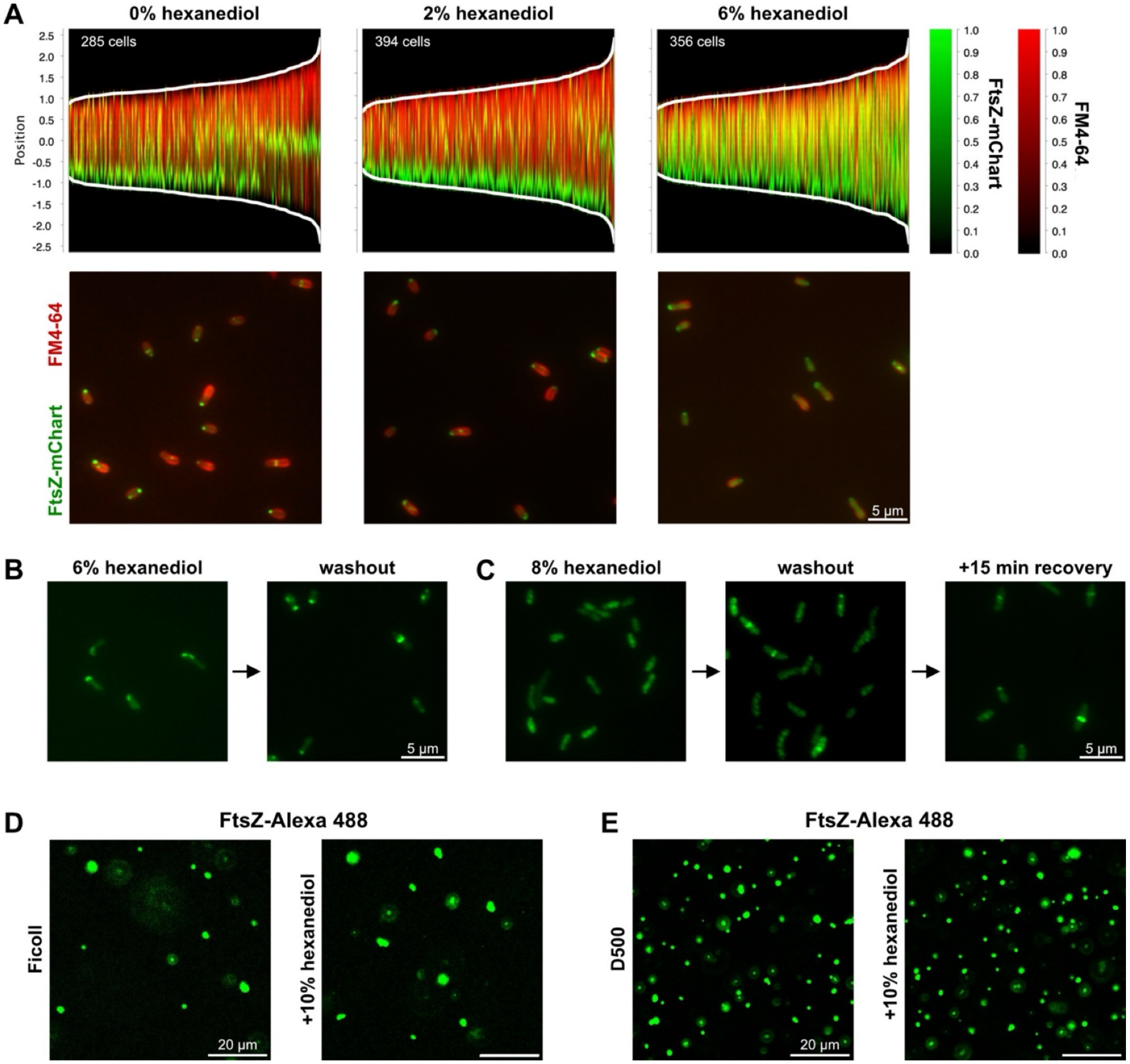

Fig S6 Hexanediol treatment disrupts $FtsZ_{At}$ foci in vivo but not in vitro. (**A**) Demographs and representative fields of view of $FtsZ_{At}$-mChart (green) expressed in *A. tumefaciens* strain WM7687. Liquid cultures were treated with 0%, 2%, or 6% 1,6-hexanediol for 15 min, then mounted on 2% agarose pads prepared with M9-glucose and the same concentration of 1,6-hexanediol. Cells were concurrently stained with the membrane dye FM4-64 (red) to distinguish old and new poles. Midcell FtsZ localization was disrupted at lower 1,6-hexanediol concentrations, whereas polar localization became disrupted at 6% or higher concentrations. (**B**) FtsZ localization in cells treated with 6% 1,6-hexanediol recovered after cells were washed with fresh media. (**C**) Cells similarly treated with 8% 1,6-hexanediol required additional recovery time to restore typical localization patterns. (**D and E**) Addition of 10% 1,6-hexanediol to in vitro condensates preformed in (**D**) Ficoll or (**E**) D500 did not result in disruption of condensate droplets. Condensate experiments were prepared as described for Fig. 1.

Table S1. Strains and plasmids used in this study

Strain	*Description*	*Source/Reference*
C43	*E. coli* C43(DE3) protein expression strain	Lab collection
C58	*A. tumefaciens* C58 wild-type	PC Zambryski
EG444	*C. crescentus xylX*:: P_{xyl}-*ftsZ-yfp*	(1)
SN1187	*E. coli* MG1655 *ΔhsdR ΔendA ΔrecA*	(2)
WM1074	*E. coli* MG1655 *ΔlacU169*	Lab collection
WM2754	*E. coli* WM1074 + pKG116-FtsZ-GFP, cm^R	Lab collection
WM7469	*E. coli* C43 + pHYRSF53-His-SUMO-$FtsZ_{At}$	this study
WM7687	*A. tumefaciens* C58 + pSRK-km-$FtsZ_{At}$-mChartreuse	this study
WM7689	*E. coli* WM1074 + pSRK-km-$FtsZ_{At}$-mChartreuse	this study
WM7692	*E. coli* WM1074 + pSRK-km-$FtsZ_{At}$-mChartreuse + pKG110-mLychee-$PopZ_{At}$	this study
Plasmid	***Description***	***Source/Reference***
pHYRSF53	6xHis-Sumo tag expression vector, km^R	(3)
pJZ207	pSRK-Km-$FtsZ_{At}$-GFP, km^R	(4)
pKG110	pACYC184 derivative with *nahG* promoter, cm^R	JS Parkinson
pKG116	pKG110 derivative with stronger Shine-Dalgarno sequence, cm^R	JS Parkinson
pWM7469	pHYRSF53-His-SUMO-$FtsZ_{At}$	this study
pWM7613	pNF02-mChartreuse (green)	(5)
pWM7615	pNF02-mLychee (red)	(5)
pWM7685	pSRK-Km-$FtsZ_{At}$-mChartreuse	this study
pWM7690	pKG110-mLychee	this study
pWM7691	pKG110-mLychee-$PopZ_{At}$	this study
pWM7714	pSRK-Km-$FtsZ_{At}$(Δ364-548)-mChartreuse	this study

Table S2. Primers used in this study

Primer	*Description*	*Sequence*
2826	IVEC pHYRSF53 fwd	taagcttgcggccgcactcg
2827	IVEC pHYRSF53 rvs	ggatccaccaatctgttctctgtgagtc
2828	IVEC Agro FtsZ fwd	tcacagagaacagattggtggatccATGACGATACAGCTGCAAAAGC
2829	IVEC Agro FtsZ rvs	agactcgagtgcggccgcaagcttaGTTGGACTGGCGGCGCAGGA
2979	pJZ207 SacI fwd	CACCATgagctcGTTGGACTGGCGGCGCAGGAAG

2980	pJZ207 HindIII rvs	AGGTAAaagcttATCGATACCGTCGACCTCGAGG
2985	mChart SacI fwd	ATGACgagctcATGTCTAAAGGTGAAGAACTG
2986	mChart HindIII rvs	GATTCCaagcttTTATTTGTAaAGCTCATCCATG
3012	mLychee PstI fwd	ATCGTGctgcagTATGGATTCAACAGAAGCAAT
3013	mLychee SpeI rvs	CACGATactagtCTTGTACAAACTTCCGCCAG
3014	PopZ KpnI fwd	ATCGTCggtaccATGGCTCAGCCAAGTGTCGC
3015	PopZ BamHI rvs	CACGATggatccTTAGCGGCGCGAGCCGCGCGC
3098	FtsZ del364-548 fwd	CGTCGTGGCCAGCTTGAC
3099	FtsZ del364-548 rvs	CTGCGATACTGCATGTGCAG

Supplemental References

1. Meier EL, Razavi S, Inoue T, Goley ED. 2016. A novel membrane anchor for FtsZ is linked to cell wall hydrolysis in *Caulobacter crescentus*. 2. Mol Microbiol 101:265–280.

2. Nozaki Shingo, Niki Hironori. 2019. Exonuclease III (XthA) enforces *In Vivo* DNA cloning of *Escherichia coli* to create cohesive ends. J Bacteriol 201:10.1128/jb.00660-18.

3. Guerrero F, Ciragan A, Iwaï H. 2015. Tandem SUMO fusion vectors for improving soluble protein expression and purification. Protein Expression Purif 116:42–49.

4. Zupan JR, Cameron TA, Anderson-Furgeson J, Zambryski PC. 2013. Dynamic FtsA and FtsZ localization and outer membrane alterations during polar growth and cell division in *Agrobacterium tumefaciens*. 22. Proc Natl Acad Sci 110:9060–9065.

5. Fraikin N, Couturier A, Mercier R, Lesterlin C. 2025. A palette of bright and photostable monomeric fluorescent proteins for bacterial time-lapse imaging. Sci Adv 11:eads6201.
